# Resilience does not explain the dissociation between chronic pain and physical activity in South Africans living with HIV

**DOI:** 10.7717/peerj.2464

**Published:** 2016-09-13

**Authors:** Antonia L. Wadley, Duncan Mitchell, Peter R. Kamerman

**Affiliations:** Brain Function Research Group, School of Physiology, Faculty of Health Sciences, University of the Witwatersrand, Johannesburg, South Africa

**Keywords:** HIV, Pain, Function, Activity, Resilience, Accelerometer, Actigraphy

## Abstract

Pain burden is high in people living with HIV (PLWH), but the effect of this pain on functionality is equivocal. Resilience, the ability to cope with adversity, may promote adaptation to pain, so we hypothesised that higher resilience would correlate with less pain-related impairment of activity. We recruited 197 black South African PLWH, 99 with chronic pain (CP) and 98 patients without. We measured pain intensity and interference using the Brief Pain Inventory, and resilience using the Resilience Scale. Participants were generally highly resilient. Greater resilience correlated with better health-related quality of life, but not with pain intensity or interference. We also measured physical activity objectively, by actigraphy, in a subset of patients (37 with chronic pain and 31 without chronic pain), who wore accelerometers for two weeks. There was no difference in duration or intensity of activity between those with and without pain, and activity was not associated with resilience. In this sample, pain was not associated with altered physical activity. Resilience did not explain differences in pain intensity or pain interference but was associated with improved quality of life. Financial stresses and the fear of HIV stigma may have driven patients to conceal pain and to suppress its expected impairment of activity.

## Introduction

It seems self-evident, especially to the patients themselves (Riley, Ahern & Follick, 1988), that chronic pain, especially if it is moderate to severe, will interfere with activities of daily living. Pain is experienced by 50–75% (depending on cohort) of people living with HIV (PLWH), and for most of them the pain is moderate to severe in intensity (Parker, Stein & Jelsma, 2014). As expected, PLWH in the US and Denmark reported significant pain-related functional impairment (Breitbart et al., 1996; Frich & Borgbjerg, 2000; Merlin et al., 2013), yet African PLWH with similar levels of pain reported little or no functional interference (Mphahlele, Mitchell & Kamerman, 2012; Voss et al., 2007; Wahab & Salami, 2011). Moderate to severe pain limits function in other populations including those with rheumatoid arthritis and dysmenorrhea (Chantler, Mitchell & Fuller, 2009; Prioreschi et al., 2013). The reasons for the apparent lack of functional interference in African PLWH and HIV-related pain are unclear, but are likely to be informed by local psychological, social, cultural and economic factors. Reports of functional interference in PLWH have been based on subjective reports previously, rather than objective measurement, which limits interpretation.

Psychosocial factors (e.g., depression, anxiety and pain catastrophizing) are associated with greater pain intensity, disability and poorer outcomes in chronic pain populations (McWilliams, Goodwin & Cox, 2004; Wertli et al., 2014; Westman et al., 2011). However, the positive attribute of resilience, the ability to overcome and adapt to adversity (Windle, Bennett & Noyes, 2011), may temper these negative psychosocial factors and increase adaptation to chronic pain (Ruiz-Párraga et al., 2015; Ruiz-Párraga, López-Martínez & Gómez-Pérez, 2012). For example, resilience was associated with increased functioning and lower impairment in patients with chronic spinal pain (Ruiz-Párraga et al., 2015). Whilst the construct of resilience has been well explored in some clinical conditions and populations, resilience and chronic pain has been less well covered (for review: Sturgeon & Zautra (2013)), and resilience and HIV even less so. Additionally, to our knowledge, there is no literature on resilience in chronic pain and HIV cohorts from Africa. Indeed, neither resilience alone, nor its impact on function, has ever been measured in individuals with HIV-related pain. Though we have suggested that resilience is high in South African PLWH (Mphahlele, Mitchell & Kamerman, 2012), we do not know for sure that this is the case and if it is resilience that accounts for the apparent lack of functional interference in the face of pain compared to cohorts in Denmark and the USA. We hypothesised that greater resilience in individuals with HIV-related pain would associate with lower pain intensity and better quality of life, and that these benefits would be reflected in greater levels of daily activity. Therefore, in the study we report here, we investigated whether resilience could account for the dissociation between chronic pain and functional impairment in South African PLWH. In addressing this question, we also report the first use of actigraphy to measure pain-related functional impairment in HIV-related individuals objectively, to determine to what extent pain interferes with function. We also validated two English-language resilience scales in isiZulu.

## Methods

**Figure 1:**
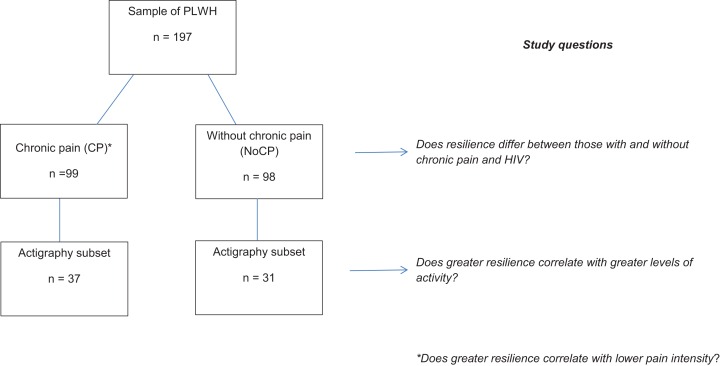
Recruitment and sample sizes for each group.

Our study was cross-sectional, and included PLWH with CP and without chronic pain (NoCP). A convenience sample of patients was recruited from the HIV clinic at Charlotte Maxeke Johannesburg Academic Hospital, South Africa, between September 2014 and March 2015. Patients waiting in the clinic queue were given a brief description of the study and if interested were invited to the study room to be given more detailed information before deciding whether to participate. Inclusion criteria were having had an HIV diagnosis for at least one year and being over 18 years of age. To be classified as having chronic pain, patients needed to report having had pain on most days of the week for at least the past three months preceding recruitment (Merskey & Bogduk, 2002) and this was established following recruitment. The NoCP group thus technically could have an acute episode of pain but by definition, this would had to have been pain experienced for less than three months. We aimed to recruit roughly the same number of patients with and without pain for a balanced design. We obtained relevant medical history from the patients’ medical records. Ethical approval was obtained from the Human Research Ethics Committee (Medical) of the University of the Witwatersrand (clearance no: M140538). Two interpreters fluent in English and local African languages helped ensure that consent was properly informed and completed data collection with patients not wishing to speak English. Figure 1 shows recruitment to each arm and sample sizes.

### Part one: questionnaires

All participants completed the following questionnaires.

The Euroqol-5D-3L (EQ5D) measures health-related quality of life (Brooks, 1996; The EuroQol Group, 1990). First, participants rated five quality-of-life domains (mobility, self-care, usual activities, pain/discomfort and anxiety/depression) on an ordinal scale (“no problems,” “moderate problems,” or “severe problems”). Second, participants rated their overall perceived state of health on a 100-point visual analogue scale (VAS; anchored at 0: “the worst health imaginable,” and 100: “the best state of health imaginable”). The EQ5D has been used widely, including in South African HIV populations (for review: Robberstad & Olsen (2010)) and has good convergent and concurrent validity, and test-retest reliability (Brazier et al., 2004; Stavem, Frøland & Hellum, 2005; van Agt et al., 1994). Internal consistency of the score is acceptable: two recent studies have reported Cronbach alphas of 0.72 and 0.73 in US multiple sclerosis caregivers and Indian patients with acute respiratory distress syndrome (Joshi et al., 2016; Shah et al., 2016).

Resilience was measured using the Resilience Scale (Wagnild & Young, 1993) and the CD-RISC (Connor & Davidson, 2003), which both have good psychometric properties including good internal consistency (Cronbach alphas = 0.91 and 0.89, respectively) and construct and convergent validity (Wagnild & Young, 1993; Connor & Davidson, 2003; Ahern et al., 2006). The Resilience Scale is a 25-item questionnaire, with each item measured on a seven-point Likert scale anchored at 1 (“strongly disagree”) and 7 (“strongly agree”). Maximum resilience scores 175. The CD-RISC also is a 25-item questionnaire, with each item measured on a five-point scale anchored at 0 (“not true at all”) and 4 (“true nearly all the time”). Maximum resilience scores 100. All participants completed both questionnaires, with the questionnaires administered in random order.

Resilience scales have not been employed previously in populations with HIV-related pain, but the Resilience Scale has been validated in a chronic pain population (Ruiz-Párraga et al., 2015; Ruiz-Párraga, López-Martínez & Gómez-Pérez, 2012), and the CD-RISC has been validated in South African cohorts, including patients with HIV infection (Jorgensen & Seedat, 2008; Spies & Seedat, 2014). We had no a priori reason to prefer one of the scales so decided to use both, and to compare their utility and outcomes. Patients were given the choice of answering the questionnaires in English or in isiZulu, the language spoken most widely by black South Africans. With the permission and input of the authors of both scales, we used a reputable national company to translate the questionnaires into isiZulu using two forward and back translations, followed by a consensus meeting. To assess the psychometric properties of the isiZulu translations, we investigated the internal consistency and factor structure of the translated questionnaires (Supplemental Material).

In addition to assessing quality of life and resilience, we also assessed resource prioritisation amongst participants (Uwimana & Struthers, 2007) by asking them how much they worried about specific life stressors: health, money, availability of food, and their family. We recorded how frequently they worried about these stressors using an ordinal scale (“not at all,” “rarely,” “sometimes,” “often,” “nearly all the time”). CP patients also were asked about any additional worries they had, and to whom they had revealed their pain; their unstructured responses were recorded.

Patients reporting chronic pain also completed The Brief Pain Inventory (BPI), an instrument used frequently to assess pain intensity and pain interference with life functions (Cleeland & Ryan, 1994). The pain intensity component of the instrument records pain intensity at its worst and at its least in the past week, as well as at the time of assessment, on an 11-point numerical rating scale anchored at 0 (“no pain”) and 10 (“the worst pain you can imagine”). The BPI also assesses average pain in the last week, but our experience with validating the Wisconsin Brief Pain Questionnaire (a precursor of the BPI) in a population of patients with similar demographics indicated that the concept of “average pain” did not translate validly amongst isiZulu speakers, so we omitted this question (Mphahlele, Mitchell & Kamerman, 2008). The pain interference component (BPI-I) assesses interference of pain on seven activities of daily living: general activity, mood, walking ability, normal work, relations with other people, sleep and enjoyment of life. Interference is scored on an 11-point numerical rating scale anchored at 0 (“does not interfere”) and 10 (“completely interferes”). The average of the seven individual interference scores provides an overall interference score. The BPI has good validity and internal consistency (α > 0.80 for both pain intensity and interference subsections) (Cleeland & Ryan, 1994; Song et al., 2016). The BPI has been used and validated in a South African amaXhosa population, as has the closely-related Wisconsin Brief Pain Questionnaire in an amaZulu population (Mphahlele, Mitchell & Kamerman, 2008; Parker, Jelsma & Stein, 2016).

### Part two: actigraphy

All patients who completed questionnaires were asked whether they would consent to wearing an accelerometer to measure their physical activity objectively by actigraphy. We aimed to achieve balanced cohorts, with half of those wearing actigraphs having chronic pain. Previous case-control studies of activity in cohorts with pain have recruited 12–20 people per cohort (Chantler, Mitchell & Fuller, 2009; Hasenbring et al., 2006; Raijmakers et al., 2015). We were unsure how much variability there might be in activity, and so aimed to recruit ∼40 to each group. Exclusion criteria for wearing actigraphs included: having a physical disability, neurological or respiratory complaint that impeded walking, or having an infant less than a year old, because of possible interrupted sleep and increased activity at night.

We used unidirectional accelerometers (Actical Step, Mini-Mitter, Respironics, Murrysville, PA, USA) to measure physical activity; the devices have been used previously to measure activity in other South African patients in pain (Chantler, Mitchell & Fuller, 2009; Prioreschi et al., 2013). As there is variation in individual accelerometer sensitivity, we referenced each accelerometer’s output to its maximum output (Bassett, Rowlands & Trost, 2012). Activity was recorded in one minute epochs. The accelerometers, which measured 29 × 37 × 11 mm and weighed about 22 g, were fitted on a belt and worn over the right hip joint for two weeks. The accelerometers were removed for bathing, showering or swimming only.

### Analyses

Continuous parametric data are presented as mean (standard deviation, SD), and were analysed using unpaired t tests. Continuous, non-parametric data are presented as median (range), and were analysed using Wilcoxon rank sum tests. Categorical data are presented as percentages, and analysed using Fisher’s exact tests (2 × 2 tables) and Chi-square test for trend (> 2 × 2 tables). All univariate test statistics were generated using bootstrap methods using 10,000 resamples. The test statistic, 95% confidence interval and original p-value are reported. Permuted p-values (10,000 permutations) were also generated, and can be seen at the following url (Kamerman, Wadley & Mitchell, 2016).

Our validation process for the isiZulu translations of the resilience questionnaires resulted in us having to remove some questions because of poor factor loading (see below: ‘Validation of the resilience scales’). This curtailment meant that the number of statements scored in the English and isiZulu versions was not the same, and therefore the maximum questionnaire score that could be achieved differed between the two language versions. There were 23 statements in the isiZulu version of the Resilience Scale and 24 in the isiZulu CD-RISC. We normalised the scores by multyiplying the isiZulu Resilience Scale score by 1.09 (25/23) and the CD-RISC by 1.04 (25/24).

### Activity analyses

To reduce irregularities in activity while the patients were getting used to wearing an actigraph, we analysed activity only from the last seven complete days. We measured: 1) time spent active, 2) intensity of activity, and 3) endurance (time spent at a particular level of activity).

1. **Time spent active:** We calculated average activity for each successive five-minute period. Periods with mean activity < 2.5% of the accelerometer maximum were classed as ‘inactive periods’ (e.g., sleeping or resting). The number of remaining five-minute ‘active periods’ was summed over 24 h and then averaged for the seven days. Median minutes active per day were compared between the participants with and without pain using a Wilcoxon rank sum test.
2. **Intensity of activity:** Using data only from ‘active periods,’ we calculated median daily activity counts for the week, and compared the scores between participants with and without chronic pain using a Wilcoxon rank sum test. We used Spearman’s rank correlations to assess associations between median daily activity and subjective measures of pain, function and resilience.
3. **Endurance:** As the analysis of median activity would not differentiate between those who had consistent low levels of activity throughout the day from those who had short bursts of very intense activity, we added an analysis of endurance. Using data from both the active and inactive periods for the week, we calculated the time each patient spent in quartiles of their maximum recorded activity during the week (0, 1–24, 25–49, 50–74, ≥ 75% of maximum). We used a Wilcoxon rank sum test to compare time spent inactive and in each quartile between the participants with and without chronic pain, and correlated these measures of endurance with resilience scores using Spearman’s rank correlation.

### Multivariate analysis

We identified variables predicting activity using random forest analysis (a nonparametric recursive partitioning technique) (Hothorn et al., 2006; Strobl et al., 2008; Strobl et al., 2007). This method was chosen as the data did not conform to the underlying assumptions of linear regression, despite attempts to transform it. Random forest analysis is designed to reduce a large set of potentially-predictive variables to only those that are informative. To assess stability of the modelling solution, we repeated each model four times, changing the number of bootstrap samples, and the number used to seed the modelling process. In all models, the number of randomly pre-selected predictor variables for each split in the tree was set at three, and the calculation of variable importance assumed possible correlation between predictors (i.e. that they were not necessarily independent variables). Variables were judged to be informative if their importance value was above the absolute value of the lowest negative-scoring variable.

We assessed for moderator effects of resilience on the associations between pain and activity using linear regression, and pain and quality of life using beta regression. Interactions were also examined in centred data using simple slope analysis.

### Validation of the resilience scales

As the data arising from the resilience scales were ordinal integers, we used polychoric correlation matrices, rather than Pearson’s correlation matrices, in the assessment of internal consistency and parallel analysis for factor number estimates.

Factor analysis was used to determine the number of distinct factors in the isiZulu versions of each of the two resilience scales, and how well the questions loaded onto each of the factors. We determined the initial number of factors to include in the analyses using the parallel analysis method proposed by Horn (1965) (Revelle, 2015). Oblique Oblimin rotation was used in factor analyses because of the likelihood of correlation between factors (Beavers et al., 2013). The factor analysis was performed iteratively, with questions with factor loadings less than 0.3 being removed successively, until all loadings in re-runs exceeded 0.3 (Beavers et al., 2013).

Our data revealed good correlation between the scores of the Resilience Scale and the CD-RISC in our sample of patients (Spearman’s correlation: r = 0.54 and p < 0.0001). We therefore picked one, the resilience scale, to use in the univariate and multivariate analyses, on the grounds that it has been used previously to measure resilience in patients with CP (Ruiz-Párraga et al., 2015; Ruiz-Párraga, López-Martínez & Gómez-Pérez, 2012; Ramírez-Maestre & Esteve, 2014; Ramírez-Maestre, Esteve & López, 2012).

We analysed our data using GraphPad Prism 4 (GraphPad, California), and R version 3.1.2 (Core R Team, 2015), using the ‘party’ package (Hothorn et al., 2006; Strobl et al., 2008; Strobl et al., 2007; Hothorn, Hornik & Zeileis, 2006) and ‘psych’ package (Revelle, 2015). Full data sets, analysis scripts, and analysis outputs for the factor, activity, and random forest analyses are available (Kamerman, Wadley & Mitchell, 2016). The site also contains the data and scripts used to generate the Supplemental Figures.

## Results

One hundred and ninety-seven black African patients were recruited to the questionnaire arm. Of those patients, 99 had chronic pain and 98 did not. Five individuals in the NoCP group reported acute pain episodes on the day of assessment. Basic demographic data for the CP and NoCP groups are shown in Table 1. In brief, the sample was primarily female (72%) and 41% had completed secondary school (12 years of schooling). Patients in pain were older, less educated, and less likely to be employed than were patients without pain (Table 1). Of the unemployed, approximately one-third in both the CP and NoCP groups were receiving a social grant (child support, disability or older persons grant). Thus 70% (69/99) and 79% (77/98) of the CP and NoCP groups respectively were receiving some form of income. The number of patients receiving a disability grant (five) was the same in both CP and NoCP groups. Recent CD4 T-cell counts were not available for the majority of the sample, but nadir CD4 T-cell counts were similar between the groups with and without pain, as was the proportion of each group with an undetectable viral load at the time of recruitment. Quality of life was significantly lower for those with pain. Indeed, patients in the CP group reported significantly greater dysfunction on each of the five EQ5D subscales. Apart from the pain subscale, the compromise for the CP group was most distinct in the mobility subscale, where 40% of the patients in the CP group, but only 6% of those in the NoCP group had some problem with mobility. The perceived overall health score for the CP group (mean 50/100) was significantly lower than that for the NoCP group (mean 80/100).

**Table 1:** Demographic characteristics of the participants.

Characteristic^*^	Chronic pain (n = 99)	No chronic pain (n = 98)	p-value
Age (years)	44 (10)	40 (10)	**0.01**^†^
Female (n (%))	65 (66)	76 (78)	0.08^‡^
Highest level of education (n (%))			**0.01**^‡^
No education	23 (23)	6 (6)	
Primary	41 (41)	44 (45)	
Secondary	28 (28)	38 (39)	
Tertiary	7 (7)	7 (7)	
Employment status (n (%))^‖^			**0.03**^‡^
Full time	37 (37)	53 (54)	
Part time/piece work	18 (18)	13 (13)	
Unemployed	44 (44)	32 (33)	
Years since diagnosis	6 (1–25)	6 (1–20)	0.92^§^
Years on antiretroviral therapy (ART)	4 (0–24)	4 (0–18)	0.57^§^
Nadir CD4 T-cell count (cells.mm^−3^)	130 (3–775)	145 (1–678)	0.98^§^
Number with undetectable viral load	77 (78)	76 (78)	0.56°
Number receiving a social grant	22 (22)	17 (17)	0.48°
Number receiving a disability grant	5 (5)	5 (5)	1.00°
***EQ5D subscales***			
*Mobility*			
No problems	59	92	**< 0.0001**^‡^
Some problems	40	6	
Unable	0	0	
*Self-care*			
No problems	94	98	**0.02**^‡^
Some problems	5	0	
Unable	0	0	
*Usual activities*			
No problems	58	94	**< 0.0001**^‡^
Some problems	40	4	
Unable	1	0	
*Pain/discomfort*			
No problems	6	93	**< 0.0001**^‡^
Moderate problems	71	4	
Extreme problems	22	1	
*Anxiety/depression*			
None	74	89	**0.002**^‡^
Moderate	22	9	
Extreme	3	0	
*EQ5D VAS*	50 (0–90)	80 (30–100)	**< 0.0001**^§^

**Notes:**

P values in bold are significant.

* Age: mean (SD). Female, Highest level of education, Employment status, Undetectable viral load, Number receiving a social or disability grant and EQ5D subscales: count (percent group size). Time since diagnosis, Time on ART, Nadir CD4 T-cell count, EQ5D VAS: median (range). Social grants include child support, older person and disability grants.

† t-test.

‡ Chi-square test for trend.

§ Wilcoxon rank sum test.

° Fishers exact.

‖ Missing education data from one individual with chronic pain and three without chronic pain.

Characteristics of the pain in the CP group are shown in Table S1. Half the CP group had developed persistent pain following their HIV diagnosis, and the average duration of the pain for the CP group was four years. Pain was experienced most frequently in the feet, with 75% (36/48) of those with foot pain experiencing bilateral foot pain, compatible with the presence of HIV-associated neuropathy. Worst pain was on average 8/10. Even though we had assigned them to the NoCP group based on their reports of pain at the time of recruitment, one patient in the NoCP group reported extreme problems with pain and a further four patients reported moderate problems in the last week. They did not, however, fit our criteria for chronic pain. Overall, the degree to which pain interfered with the activities of daily living in the CP group was five, on the scale 0–10. Amongst the activities of daily living, pain interfered most with sleep and with general activity, but surprisingly little with enjoyment of life.

Table 2 shows how much the CP and NoCP groups worried about their health and other stressors. A significantly greater proportion of patients in pain reported higher levels of worry for every stressor than those without pain. Worries about money were the most frequent for both CP and NoCP groups, with 84% of patients in the CP group and 55% in the NoCP group worried about money “nearly all the time.” Even though they had positive HIV diagnoses, 28% of patients in the NoCP group were not at all concerned about their health, while only 9% of patients with pain were not at all concerned. When we split the sample into employed and unemployed patients and then looked at the differences in worries between the CP and NoCP groups, the patients in pain worried about each of the life stressors more than their employment-matched pain-free counterparts (Chi-square tests for trend; p ≤ 0.02; for clarity, see Kamerman, Wadley & Mitchell, 2016). Demographic data shed further light on the economic stresses of the whole sample (n = 197): 21 patients (11%) came from households where nobody was earning an income, and of the remaining households, there were on average two dependents for each earner (range 0–11). When additional dependents living outside the home were taken into account, the average was three dependents per earner (range 0–11).

**Table 2:** Frequency of worries about life stressors in the cohorts with and without chronic pain.

	Health concerns			Money			Food			Family concerns		
	CP	NoCP	p	CP	NoCP	p	CP	NoCP	p	CP	NoCP	p
Not at all	9 (9)	26 (27)	< 0.0001	0 (0)	7 (7)	< 0.0001	14 (14)	40 (40)	< 0.0001	7 (7)	23 (23)	< 0.0001
Rarely	2 (2)	10 (10)		1 (1)	4 (4)		5 (5)	6 (6)		2 (2)	3 (3)	
Sometimes	29 (29)	33 (34)		11 (11)	27 (28)		31 (31)	23 (23)		15 (15)	22 (22)	
Often	6 (6)	8 (8)		5 (5)	6 (6)		3 (3)	8 (8)		5 (5)	8 (8)	
Nearly all the time	53 (54)	21 (21)		82 (83)	54 (55)		46 (46)	22 (22)		70 (70)	42 (43)	

**Notes:**

Data presented as count (%).

CP, chronic pain; NoCP, group without chronic pain.

Chronic Pain n = 99; No Chronic Pain n = 98. Analysed with a chi squared test for trend.

Patients in the CP group also were asked an open question about other worries they were facing. Patients indicated that one of their other greatest worries was being “gossiped about,” that is, their HIV status being revealed and discussed by others. Indeed, 44% (44/99) had not told their friends and 9% (9/99) had not told their families about their chronic pain for fear that it would reveal their HIV status.

### Univariate analysis

The validation of the resilience scales is shown in the Supplemental Material and in Kamerman, Wadley & Mitchell (2016).

Mean scores for the resilience scales and comparisons between the CP and NoCP groups are shown in Table 3. Resilience scores were high. Participants in pain had lower resilience scale scores than did those not in pain, but even in the sub-group with least resilience, namely patients in pain who completed the questionnaires in isiZulu, the mean resilience score was not below 72% of the maximum for either scale. For all the patients without pain, resilience score exceeded 83% of maximum on both scales.

**Table 3:** Mean resilience scale scores.

	n	Questionnaire scores pain cohort	n	Questionnaire scores pain-free cohort	p
**The resilience scale**					
RS-25 in English completers	20	155 (12)	26	153 (15)	0.61
RS-23 in isiZulu completers	78	130 (18)	73	143 (12)	**< 0.0001**
Full cohorts	98	144 (19)	99	155 (13)	**< 0.0001**
**Connor-Davidson resilience scale**					
CD-RISC 25 in English completers	21	86 (8)	22	81 (16)	0.18
CD-RISC 24 in isiZulu completers	77	72 (12)	77	80 (9)	**< 0.0001**
Full cohorts	98	77 (12)	99	83 (11)	**< 0.001**

**Notes:**

P values in bold are significant.

Data presented as mean (SD). Scores analysed with an unpaired t test.

In a cohort analysis, we assessed the association between resilience scores and pain intensity, nadir CD4 T-cell count (as an index of disease severity) and subjective ratings of functional interference in the group CP (n = 99). Resilience did not correlate with either worst (Spearman’s correlation; r = 0.183, 95% CI −0.01–0.38, p = 0.07) or least pain in the last week (Spearman’s correlation; r = −0.027, 95% CI −0.25–0.19, p = 0.79). Neither did resilience correlate with nadir CD4 T-cell count (Spearman’s correlation; r = −0.110, 95% CI −0.30–0.06, p = 0.30). Furthermore, resilience did not correlate with BPI-I scores, the subjective measure of functional interference (Spearman’s r = 0.099, 95% CI −0.11–0.31, p = 0.33). When comparing pain intensity with quality-of-life scores, we found worst pain did not correlate with perceived state of health (using the EQ5D VAS scale) (Spearman’s; r = −0.170, 95% CI −0.36–0.02, p = 0.09). Scores correlated well for the two subjective measures of functional limitation with mobility, that is, the mobility questions on the EQ5D and BPI interference scale (Wilcoxon rank sum; p = 0.02).

For the whole sample (n = 197), resilience was correlated with ratings of overall perception of health status (EQ5D VAS scale), with higher resilience scores associated with greater perceived health (Spearman’s; r = 0.407, 95% CI 0.29–0.54, p = < 0.0001). We looked for moderator effects of resilience on the association between pain and quality of life in the pain group (n = 99) but saw no evidence of interaction (r^2^ = 0.12, p = 0.24; for clarity, see Kamerman, Wadley & Mitchell, 2016).

### Actigraphy results

Of the 197 patients recruited to the study, 77 volunteered to wear accelerometers. Nine patients’ data were excluded from the analyses: seven because they wore the accelerometers inconsistently, one patient was lost to follow-up, and one patient’s accelerometer battery failed. Of the 68 patients included in the analysis, 37 had chronic pain and 31 did not. Demographic characteristics of the actigraphy subsets versus the remaining sample demonstrated that the subsets were representative of the larger groups with and without pain (Table S1). In particular, there was no difference in BMI between the two groups (Mean (SD): pain cohort 28 (6) kg m^−2^ vs 26 (6) kg m^−2^ pain-free cohort; t test; p = 0.09). Differences between the CP and NoCP groups included education and employment, with the CP group having completed a lower level of education and less likely to be employed (p < 0.05; for clarity, see Kamerman, Wadley & Mitchell, 2016).

We compared duration of time spent active and intensity of activity between the CP and NoCP groups. There was no difference for either component: mean time spent active each week (Wilcoxon rank sum; test statistic 0.265, 95% CI −1.69–2.23, p = 0.89) or median daily activity counts (as a percentage of the accelerometer’s calibrated maximum) (Wilcoxon rank sum; test statistic −0.895, 95% CI −2.85–0.10, p = 0.38) (Fig. S1).

**Figure 2:**
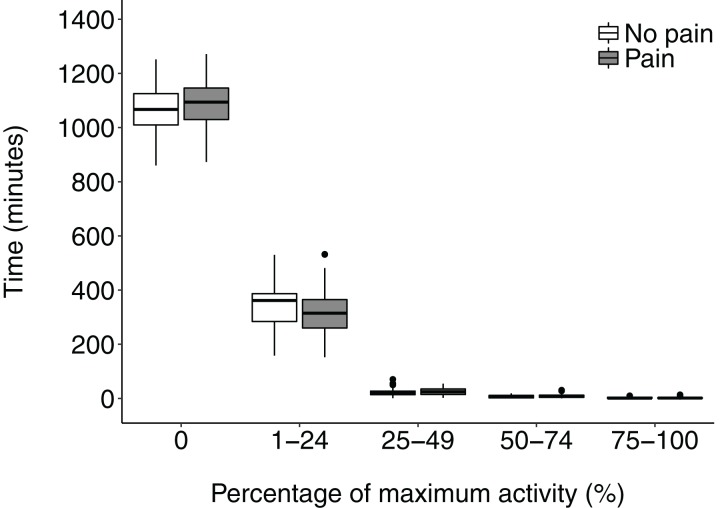
Time spent by patients with and without chronic pain in different quartiles of activity intensity. Data analysed with a Wilcoxon rank sum test.

Having calculated each patient’s maximum activity count, we assessed time spent at 0, 0–24, 25–49, 50–74, and > 75% of maximum activity. There was no difference in time spent at 0% and in each of the quartiles between those with CP and those not (Wilcoxon rank sum; p values for each quartile > 0.05) (Fig. 2).

### Univariate analysis

Figure S2 shows correlations between resilience and activity. Resilience scores did not correlate with median activity in the CP (Spearman’s r = −0.182, 95% CI −0.54–0.14, p = 0.42) or NoCP groups (Spearman’s r = 0.024, 95% CI −0.35–0.40, p = 0.94). However, for patients CP, resilience correlated inversely with time spent active, within the 25–49 and 50–74% quartiles of activity intensity (Spearman’s r = −0.460; 95% CI −0.78 to −0.21, p = 0.005 and Spearman’s r = −0.397, 95% CI −0.73 to −0.15, p = 0.01 respectively), but the association was weak. There was no correlation between resilience and time spent at zero activity or with time spent active within the > 75% activity quartile, and no relationship between resilience and time spent active, within any quartiles of activity intensity, for those not in chronic pain (Spearman’s correlations; p > 0.05). We assessed for moderator effects of resilience on pain and activity and saw no evidence of interaction (r^2^ = 0.11, p = 0.27 ; for clarity see url (Kamerman, Wadley & Mitchell, 2016)).

Figure S3 shows associations between factors associated with activity levels in the chronic pain group only (n = 37). Neither intensity nor duration of activity correlated with subjective measures of functional interference including the walking scale of the BPI -I (Intensity of activity: Spearman’s r = 0.071, 95% CI −0.39–0.23, p = 0.58; Duration of activity: Spearman’s r = 0.042, 95% CI −0.36–0.26), p = 0.76) or the mobility scale of the EQ5D (Intensity of activity: Mann Whitney, p = 0.34; Duration of activity: Mann Whitney, p = 0.79). Furthermore, neither worst nor least pain intensity associated significantly with activity (Spearman’s correlation; r = −0.088, 95% CI −0.44–0.25, p = 0.61 and r = −0.058, 95% CI −0.40–0.28, p = 0.73, respectively).

### Multivariate analysis

**Figure 3:**
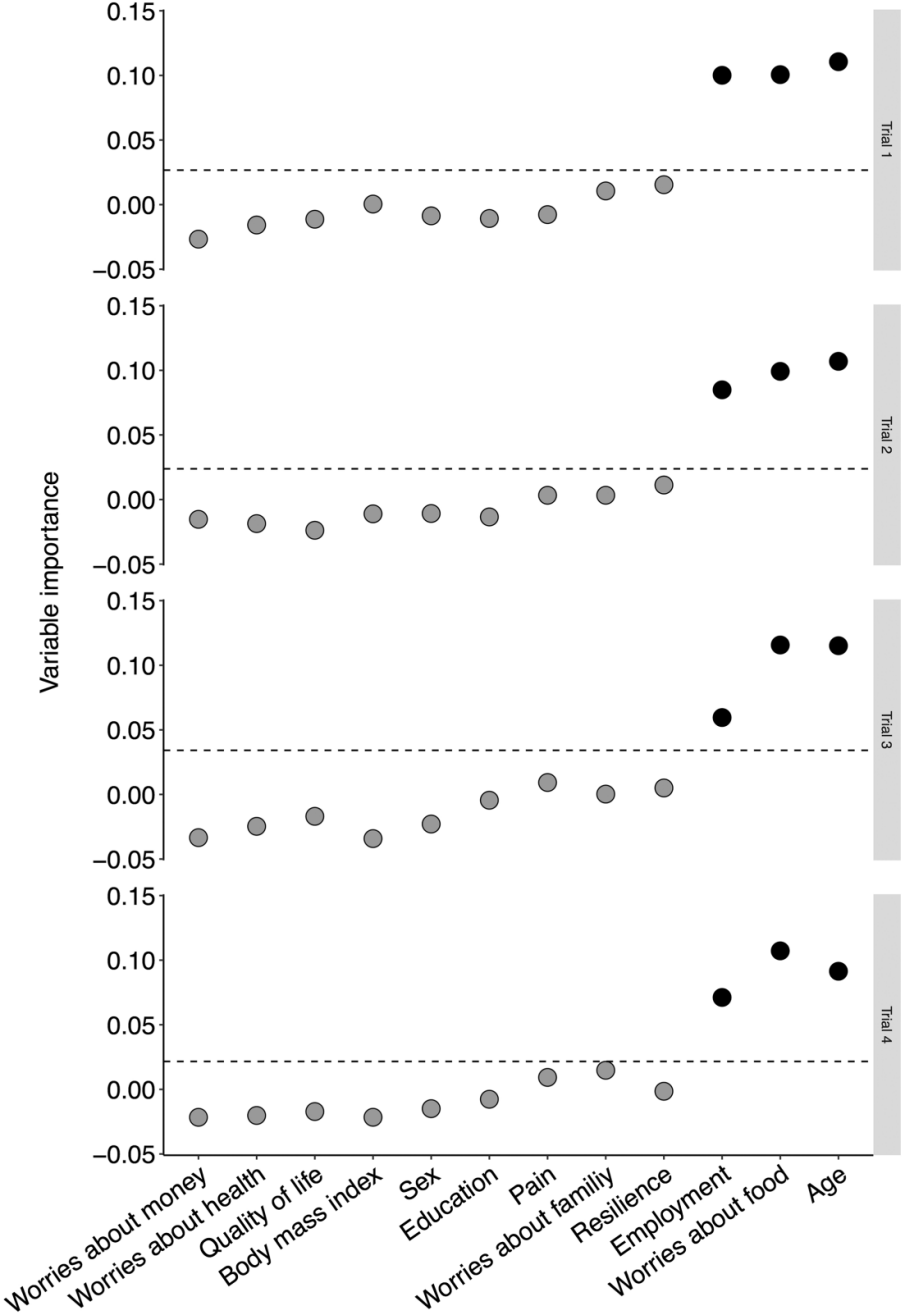
Random forest plot of predictors of activity under four modelling conditions. Predictors above the dashed line are informative variables for predicting activity.

We used a random forest analysis to explore independent predictors of activity. As pain did not associate with any of the measures of activity, we collapsed the groups and completed the analysis on the full sample of actigraphy patients (n = 68). Lower age, being unemployed and more frequent worries about food consistently had variable importance rating greater than the threshold (Fig. 3). Neither pain nor any of the other remaining factors was associated with level of activity. We assessed for moderator effects of resilience on association between pain and activity but saw no evidence of interaction (r^2^ = 0.11, p = 0.27).

## Discussion

Previously we have reported low pain-related functional interference in black South Africans living with HIV and who had moderate-to-severe pain (Mphahlele, Mitchell & Kamerman, 2012). What we then called “stoicism” also was evident in other populations of PLWH in sub-Saharan Africa (Voss et al., 2007; Wahab & Salami, 2011). The phenomenon not only contradicted the usual association between chronic pain and functional impairment (Chantler, Mitchell & Fuller, 2009; Dansie et al., 2014; van den Berg-Emons et al., 2007) but was in stark contrast with data from PLWH in the US and Denmark (Breitbart et al., 1996; Frich & Borgbjerg, 2000; Merlin et al., 2013), and even India (Nair et al., 2009), in which there was significant impairment in the face of similar levels of pain. What was absent, though, was any formal assessment of stoicism, or what is better called “resilience,” in PLWH in pain, or investigation of whether such resilience could account for the dissociation between pain and function. Although physical activity has been measured by actigraphy in PLWH patients previously (Fillipas et al., 2010), no-one has measured objectively the impact of pain on physical activity in PLWH. So we measured resilience, pain and quality of life in black South Africans with chronic HIV-related pain. Second, we investigated the degree to which chronic HIV-related pain was associated with functional impairment, including impairment measured objectively by actigraphy in a subset of patients, and whether resilience modulated the relationship between pain and activity levels. During the process, we also validated isiZulu versions of the resilience scale and CD-RISC, and came to some conclusions about the psyche of the patients that might help explain their behaviour under adversity. Ours is the first study to assess resilience in a cohort with HIV-related pain, and also the first to report objective activity data in such a cohort. Higher resilience was associated positively with better quality of life, but we found no association between resilience and pain.

It is not just in patients with HIV-related pain that resilience has not been measured previously; measuring resilience in chronic pain cohorts is a relatively new concept. We used two resilience scales, translated both into isiZulu, and found good correlation in the resilience scores delivered by the two scales in their isiZulu translations (Supplemental Material). The resilience scale previously has been used in an expanding chronic pain cohort in Spain (Ruiz-Párraga et al., 2015; Ruiz-Párraga, López-Martínez & Gómez-Pérez, 2012; Ramírez-Maestre & Esteve, 2014; Ramírez-Maestre, Esteve & López, 2012), and the widely-used CD-RISC has been employed in a South African HIV cohort (Spies & Seedat, 2014), though not in relation to pain. Our mean score for resilience for the whole cohort of patients derived from the CD-RISC was similar to the score obtained for another South African HIV cohort (Spies & Seedat, 2014). That score was high, compared with the resilience scores of other pain cohorts (Ruiz-Párraga, López-Martínez & Gómez-Pérez, 2012; West et al., 2012). After adjustment for the different number of items in the questionnaire, our patients in HIV-related pain had a mean resilience score 22% higher than did the patients of the Spanish chronic pain cohort (Ruiz-Párraga, López-Martínez & Gómez-Pérez, 2012). Indeed, the resilience scores we obtained for both scales were equivalent to those obtained previously from healthy populations elsewhere (Wagnild & Young, 1993; Connor & Davidson, 2003; Peng et al., 2012). The score on the Resilience Scale for the patients without pain was about 8% higher than for the patients with pain. Whilst the difference was statistically significant, we doubt whether it was clinically significant. The important outcome was that resilience would be categorised as high in both cohorts (Wagnild & Young, 1993). It is possible that there was some lack of sensitivity with the scales as scores all fell near the upper end of the scale, demonstrating possible ceiling effects. Nevertheless, black South African PLWH exhibited high resilience, whether or not they were in pain.

High resilience was beneficial for patients. We found higher resilience to be associated with better quality of life, as has been found for another cohort of South African PLWH (Dageid & Grønlie, 2015). Resilience, however, did not explain the lack of association between pain and functional impairment in the patients here, and neither was it a moderator of the relationship. There was no association between resilience and pain intensity, or between resilience and subjective functional interference (measured using the BPI). However, we did find an association between pain intensity and subjective interference, as reported in other cohorts with HIV-related pain (Mphahlele, Mitchell & Kamerman, 2012; Hitchcock, Meyer & Gwyther, 2008). Though functional impairment was not as low as the impairment that we reported previously in patients with HIV-related pain from the same community (Mphahlele, Mitchell & Kamerman, 2012), it still was low for patients in moderate to severe pain, with mean interference scored 5/10 (Table 2).

Resilience previously has been defined as low functional interference in the face of high pain severity (Karoly & Ruehlman, 2006). Based on this definition, resilience has been associated with better adaptation to chronic pain including lower catastrophizing and better coping skills. We used a similar definition of resilience (‘the ability to cope with adversity’), which uses the construct of resilience as a trait, and we used scales that measured it as such. There is no agreed-upon definition of resilience, however, and the term can also refer to an outcome or process (Ahern et al., 2006). Our study was cross-sectional so we cannot comment on whether resilience leads to better pain coping (resilience being an adaptive trait) or whether pain led to better resilience (resilience being an outcome). Longitudinal studies are required to determine whether resilience should be interpreted as an outcome or trait in the context of chronic pain.

For the first time in patients with HIV-related pain, we assessed impairment with physical activity objectively by actigraphy in a subset of participants. Activity measured objectively in these participants did not correlate significantly with impairment measured on the BPI or EQ5D. Despite the patients reporting high levels of pain (median intensity 8/10 for worst pain in the past week) and with 40% of patients in pain reporting problems with mobility, the patients in pain who volunteered for actigraphy actually were as active as their pain-free counterparts, whether we assessed their physical activity in terms of duration of activity per day, intensity of activity or time spent in different intensity quartiles of activity. With the high levels of pain and interference reported, it is possible that these patients had poor pain acceptance (Jensen et al., 2016), yet felt forced to keep active for the social and economic reasons described here. The lack of reduction in activity in patients living with HIV and chronic pain is an important finding. Not only does it contrast with other types of chronic pain including fibromyalgia and rheumatoid arthritis (Chantler, Mitchell & Fuller, 2009; Prioreschi et al., 2013). The finding also highlights that assumptions about PLWHs’ pain intensity and suffering cannot be based on how well they seem to be coping and how active they are.

It was a limitation of our study that only 35% of the sample wore accelerometers, but not all patients were willing or able to wear an accelerometer for two weeks and then come back to the clinic to return the device. A logistically easier and less expensive way of collecting activity data would be to ask patients to wear a basic pedometer; however, we would only have been able to glean step counts rather than data on duration and intensity of activity, while still encountering the issue of patients’ willingness to wear and return the device. Future studies may consider collecting actigraphy data from participants attending community clinics close to their homes (rather than a more distant tertiary hospital), so that return visits are more possible.

There were five individuals in the NoCP group who reported acute pain on the day of assessment. The presence of acute pain is typical in a general population (Ohayon & Stingl, 2012) and we feel this made the NoCP group representative of a general population. The mean resilience scores and median EQ5D VAS scores did not differ when these five participants were omitted (Resilience scale: unpaired t test, p = 0.96; EQ5D VAS: Wilcoxon rank sum, p = 0.84). Only one of these individuals was recruited to the actigraphy arm. Median activity and median time spent active (our measures of intensity and duration of activity) did not differ when this individual was omitted (Wilcoxon rank sum tests: Median activity, p = 0.84; Median time spent active, p = 1.0).

The lack of impact of pain on physical activity was confirmed by multivariate analysis: pain was not one of the factors predicting activity (Fig. 3). Even the patients without CP seldom engaged in activity exceeding 25% of their maximum (Fig. 2), and, on average, spent 18 h out of every 24 h inactive. In some cohorts CP of other aetiology, higher levels of activity are seen as helpful (Lin et al., 2011; Patel, Dansie & Turk, 2013), but in others endurance behaviour (persistence with activity despite pain (Hasenbring & Verbunt, 2010)) increases risk of disability and chronicity (Hasenbring & Verbunt, 2010; Huijnenl et al., 2011). Because we did not have a control cohort of HIV-negative patients from the same community, we cannot judge whether being HIV-positive reduced activity, irrespective of pain, though activity in the overall sample seemed similar to that of healthy cohorts and controls in other studies in South Africa (Prioreschi et al., 2013; Cook, Alberts & Lambert, 2012).

The lack of influence of chronic pain on physical activity measured objectively, which we have reported here, is not unique to HIV-related pain. Others have found that intensity of chronic back pain did not influence activity measured objectively (Hasenbring et al., 2006; Huijnen et al., 2010). Pain did influence the well-being of patients, though, in ways not related directly to activity. Though HIV-positive, very few of the patients without chronic pain were concerned about their health (Table 3), but more than a quarter of patients with CP were concerned. We previously have raised the idea that having pain is what induces HIV-positive South Africans to conclude that they are sick (Mphahlele, Mitchell & Kamerman, 2012). PLWH in a rural community with low pain intensity presented late for treatment, and so had more-advanced disease (Mphahlele, Mitchell & Kamerman, 2012). In our sample, more patients in pain worried about life stressors, and especially about money and having sufficient food, than did pain-free patients (Table 3). Having HIV infection massively compounds the impact of poverty, on patients and their dependents, in poor South African households (Collins & Leibbrandt, 2007). As more of the patients in pain were unemployed we stratified the worries data by employment. Having taken employment out of the question, patients in pain still worried about each of the life stressors more than the pain-free patients. As our study was cross-sectional, we do not know whether having pain increased the worries, or the increased worries predisposed patients to developing pain although a recent study supports the latter (Chou, Parmar & Galinsky, 2016). Others have suggested that it is worries about money that compel African PLWH to continue with activities of daily living, in the face of pain (Uwimana & Struthers, 2007; Phaladze et al., 2005). Their worries tended to be private, though; nearly half of our patients in pain had not told their friends about the pain, for fear of their HIV status being revealed and the patient “gossiped about.” HIV stigma remains highly prevalent in South Africa (South African National AIDS Council, 2012). The inclusion of an appropriate stigma scale in future studies would help to determine the impact of HIV stigma on pain and activity in HIV populations. It is possible that the high levels of activity maintained by our patients in pain may have been used as a strategy to conceal HIV disease.

It cannot be assumed that the lack of functional interference is positive. Endurance behaviour (continuing with activity despite pain) has been associated with increased risk of chronicity and disability (Hasenbring & Verbunt, 2010) and future studies are needed to determine whether the high level of activity in PLWH with pain is adaptive or maladaptive. Like studies of other painful conditions and in an HIV cohort where pain was not measured, we also found discrepancies between subjective and objective measures of functional impairment (Fillipas et al., 2010; Huijnenl et al., 2011; Segura-Jiménez et al., 2013; van Weering, Vollenbroek-Hutten & Hermens, 2011). Such discrepancies between subjective measures of functional interference lead us to believe that future investigations of functional interference in HIV-related pain (and pain of other aetiologies) should include objective measures of activity (Lo et al., 2015).

So, contrary to our hypothesis, it was not individual resilience that accounted for the dissociation between pain and ability to cope with daily living, in these South African PLWH. That does not mean, though, that we should abandon resilience as a trait relevant to the management of patients with HIV-related pain. In these patients, resilience was a significant determinant of quality of life. In them, as in other patients living with HIV , there were many psychological and social factors that influenced quality of life more than did pain intensity (Keltner et al., 2012). HIV-related pain is notoriously difficult to treat pharmacologically (Finnerup et al., 2015; Kamerman & Mitchell, 2011), and we may not be able to offer patients with HIV-related pain guaranteed pain relief for the foreseeable future. For those with low quality of life, though, we may be able to offer guaranteed improvement to quality of life, using strategies to improve resilience.

## Supplemental Information

10.7717/peerj.2464/supp-1 Supplemental Information 1 Pain characteristics of the chronic pain group. Pain severity and pain interference as recorded from the BPI.

10.7717/peerj.2464/supp-2 Supplemental Information 2 Validation of the resilience scales.

10.7717/peerj.2464/supp-3 Supplemental Information 3 Comparison of activity between groups with and without chronic pain.

10.7717/peerj.2464/supp-4 Supplemental Information 4 Correlations between resilience and activity.

10.7717/peerj.2464/supp-5 Supplemental Information 5 Associations between activity (measured objectively with actigraphy) and pain interference and intensity (measured subjectively using the BPI walking, EQ5D mobility and BPI worst pain intensity questions). No patients reported having moderate or severe difficulties with mobility on the EQ5D.

## Additional Information and Declarations

### Data Deposition

The following information was supplied regarding data availability: Kamerman P, Wadley A, Mitchell D. Resilience 2016. HIVPain v0.1.1. doi: 10.5281/zenodo.47321. https://zenodo.org/record/47321#.VyiedTGPzjw.
