# Supplementary material for: Resilience does not explain the dissociation between chronic pain and physical activity in South Africans living with HIV
Source: PeerJ. 2016 Sep 13;4:e2464. doi: 10.7717/peerj.2464 (PMC5028784; doi:10.7717/peerj.2464)
Supplement: Supplemental Information 1 — Pain severity and pain interference as recorded from the BPI. [file peerj-04-2464-s001.docx]

|  | **Chronic pain group**  **N=99** |
| --- | --- |
| Developed pain after HIV diagnosis, n (%) | 54 (55) |
| Average duration of pain (years) | 4 (0.25-17) |
| Number of pain sites, n (range) | 2 (1-9) |
| Pain sites, n (%)  Head  Chest  Abdomen  Thoracic spine  Lumbar spine  Lower leg pain  Feet | 16 (16)  13 (13)  13 (13)  17 (17)  23 (23)  32 (32)  49 (49) |
| Pain severity, median (range)  Worst pain  Least pain  Pain at time of assessment | 8 (3-10)  3 (0-9)  3 (0-10) |
| Pain interference, median (range)  General activity  Mood  Walking  Normal work  Relations with others  Sleep  Enjoyment of life  Overall average score | 7 (0-10)  6 (0-10)  6 (0-10)  5 (0-10)  4 (0-10)  7 (0-10)  3 (0-10)  5 (0-9) |
