# Supplementary material for: Resilience does not explain the dissociation between chronic pain and physical activity in South Africans living with HIV
Source: PeerJ. 2016 Sep 13;4:e2464. doi: 10.7717/peerj.2464 (PMC5028784; doi:10.7717/peerj.2464)
Supplement: Supplemental Information 2 [file peerj-04-2464-s002.docx]

**The Resilience Scale**

One-hundred and fifty-one participants completed the Resilience Scale in isiZulu. Internal consistency was excellent (α = 0.93). Parallel plot analysis suggested a one-factor solution, which was confirmed by exploratory factor analysis with Oblimin rotation. This single factor explained 37% of the variance. Two questions had factor loadings <0.30; Q20 (“Sometimes I make myself do things whether I want to or not”) and Q11 (“I seldom wonder what the point of it all is”). These questions were removed and the analysis re-run. Subsequently all questions had factor loadings >0.30.


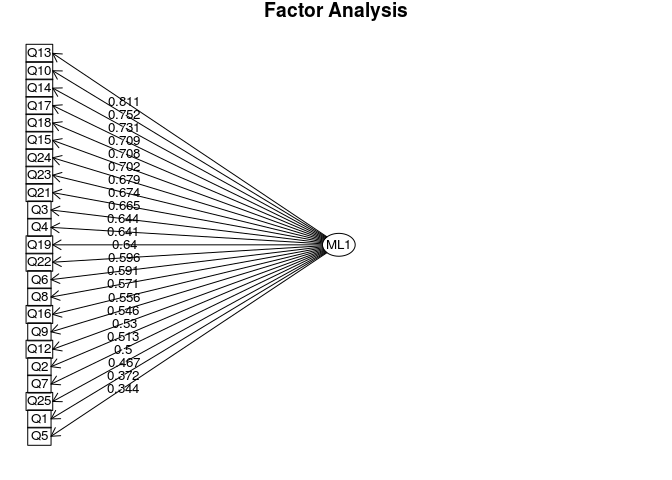


**CD-RISC**

One-hundred and fifty-four participants completed the isiZulu version of the CD-RISC. Internal consistency was excellent (α = 0.9). Parallel plot analysis suggested a three-factor solution, which was confirmed by exploratory factor analysis with Oblimin rotation. The three-factor solution explained 40% of the variance. One question had a factor loading <0.3 “I have at least one close and secure relationship that helps me when I’m stressed”. This question was removed and the analysis re-run. The subsequent analysis produced factor loadings of >0.3 for all remaining items. Two of the factors were consistent with ‘personal competence’ and ‘trusting one’s instincts’, while we labelled the third factor ‘tenacity’ (Jorgensen and Seedat 2008)

.


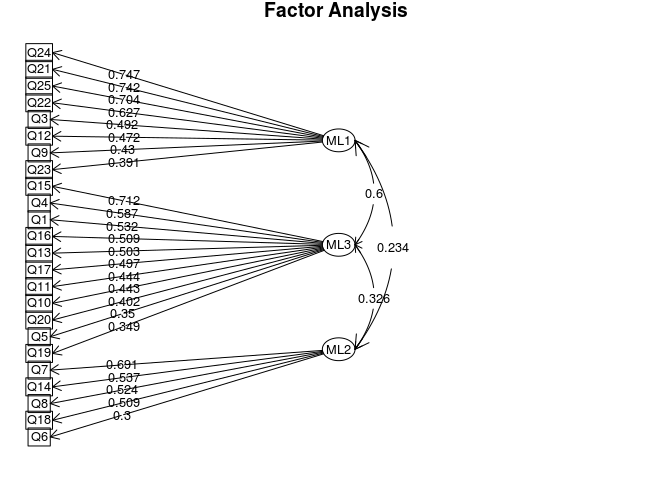


Proportional scores were calculated for those answering the RS-23 and the CD-RISC-24 so that the data could be combined with those answering the full 25-question scales in English. Using these proportional scores, the mean score for the whole cohort (n=197) was 150 (17) for The Resilience Scale and 80 (12) for the CD-RISC.
