# Supplementary figures and images for: Resilience does not explain the dissociation between chronic pain and physical activity in South Africans living with HIV
Source: PeerJ. 2016 Sep 13;4:e2464. doi: 10.7717/peerj.2464 (PMC5028784; doi:10.7717/peerj.2464)

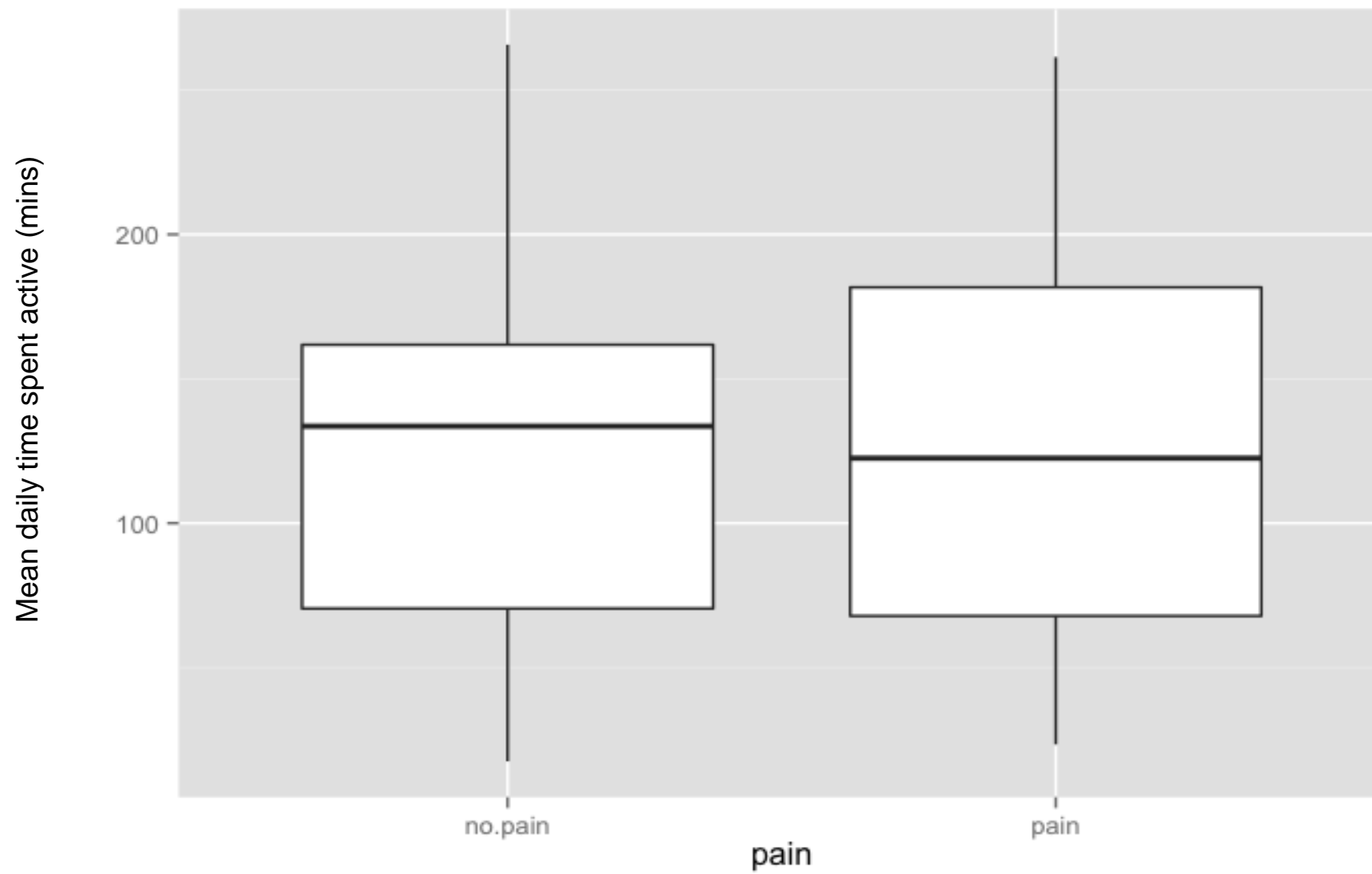

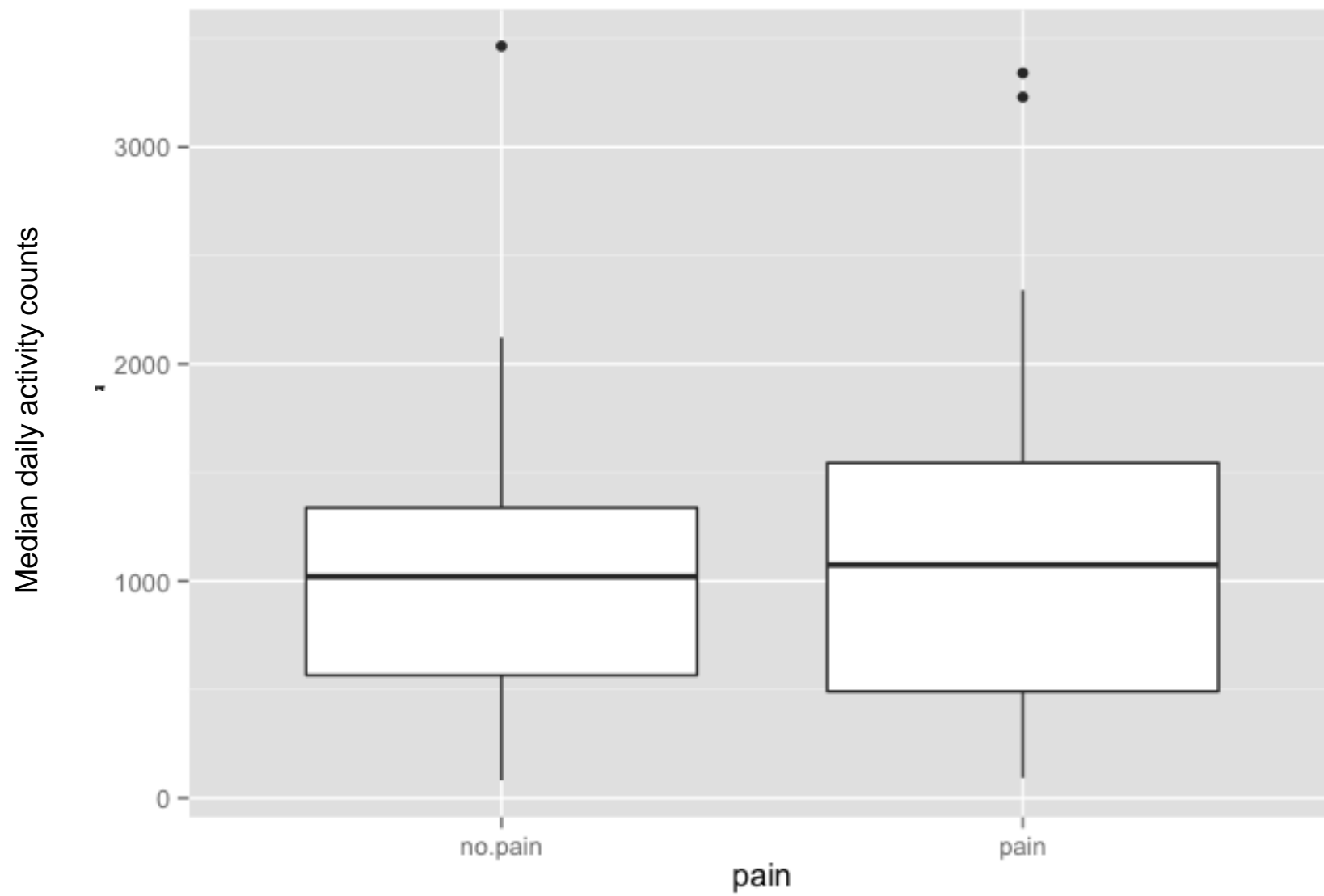

Supplement: Supplemental Information 3 [file peerj-04-2464-s003.pdf]
