# Supplementary material for: Resilience does not explain the dissociation between chronic pain and physical activity in South Africans living with HIV
Source: PeerJ. 2016 Sep 13;4:e2464. doi: 10.7717/peerj.2464 (PMC5028784; doi:10.7717/peerj.2464)
Supplement: Supplemental Information 4 [file peerj-04-2464-s004.pdf]

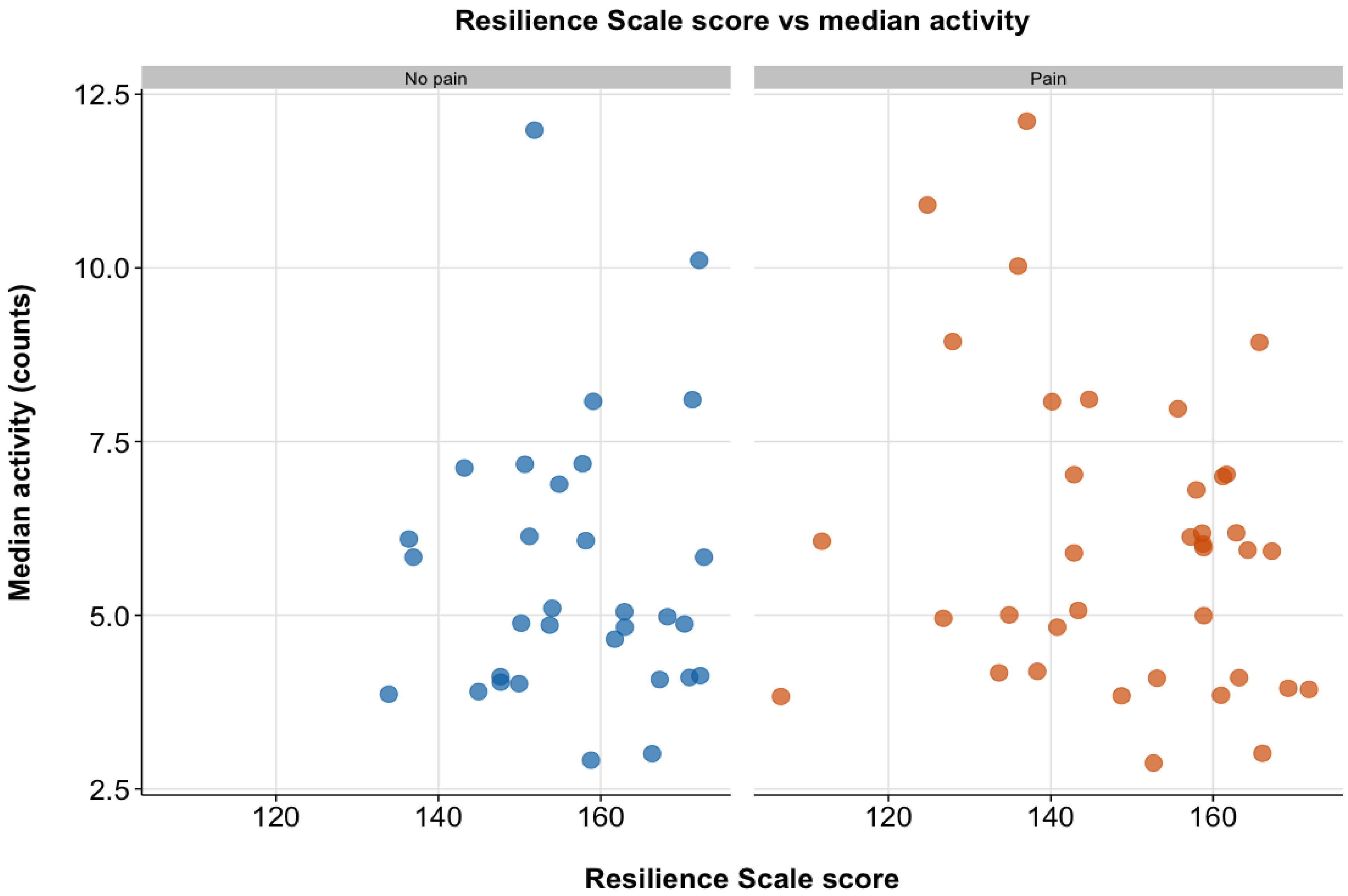

Resilience Scale score vs median activity
No pain
Pain
12.5
10.0
7.5
5.0
2.5
120
140
160
120
140
160
Median activity (counts)
Resilience Scale score

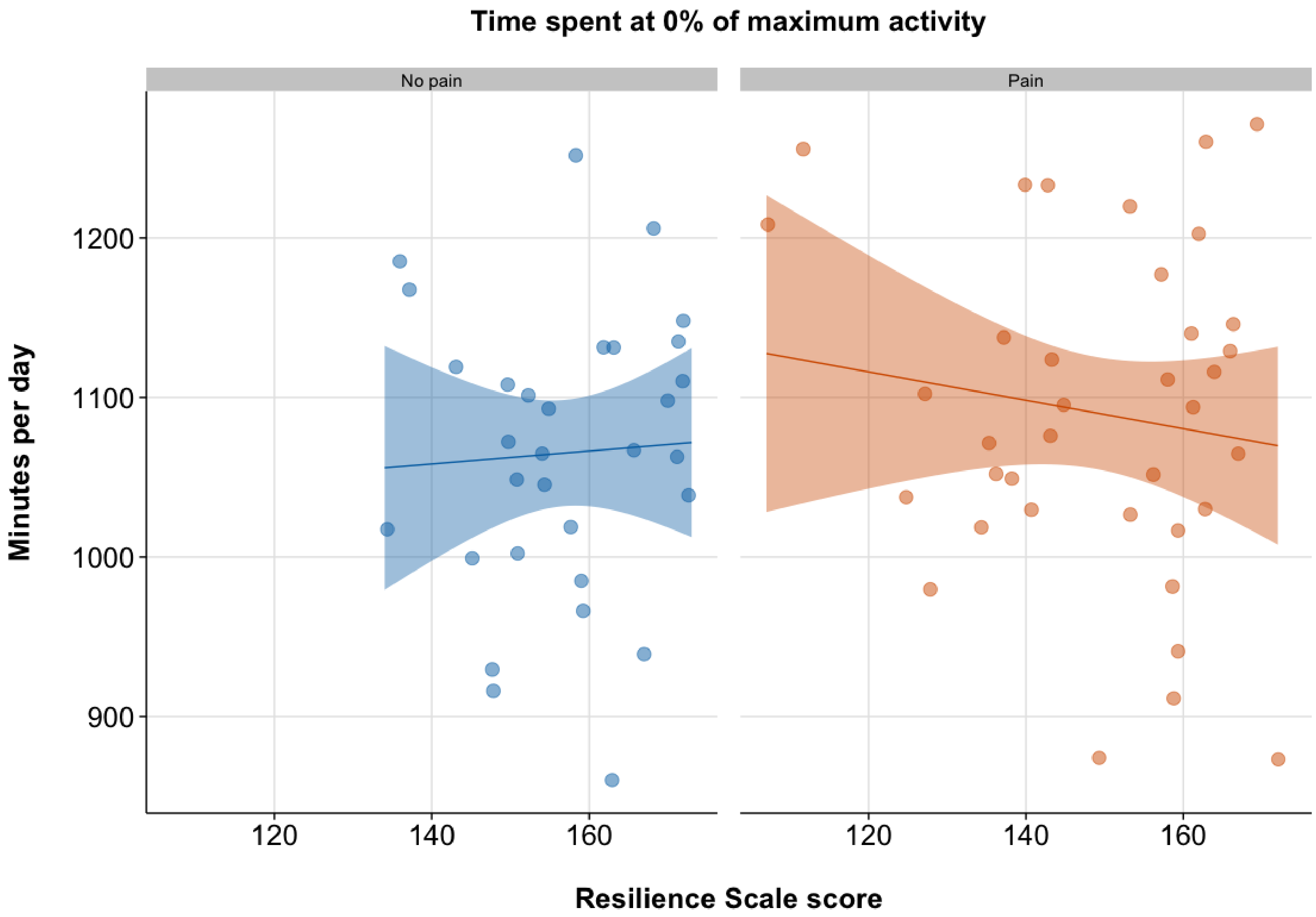

Time spent at 0% of maximum activity
No pain
Pain
Minutes per day
1200
1100
1000
900
120
140
160
Resilience Scale score

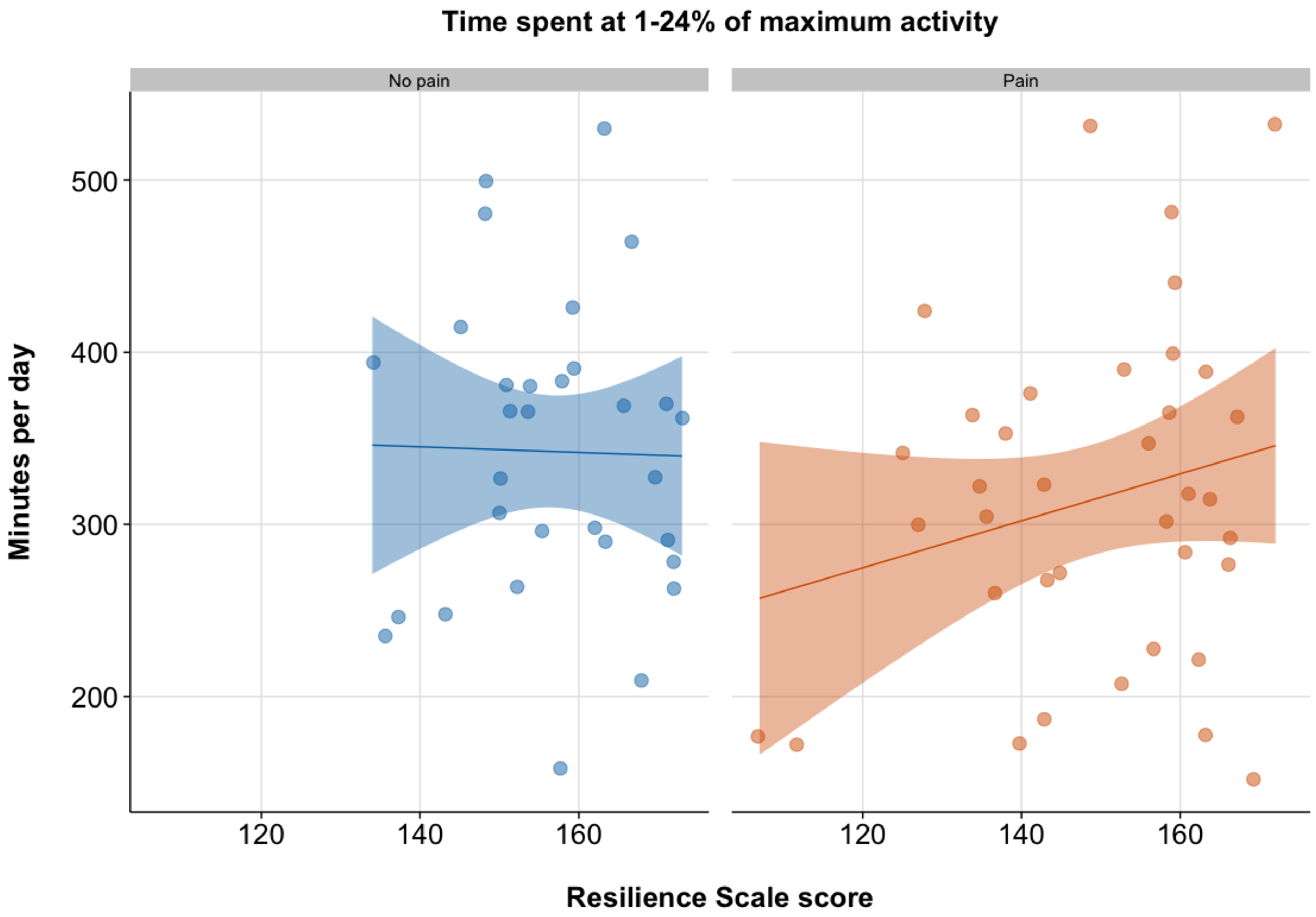

Time spent at 1-24% of maximum activity
No pain
Pain
Minutes per day
500
400
300
200
120
140
160
120
140
160
Resilience Scale score

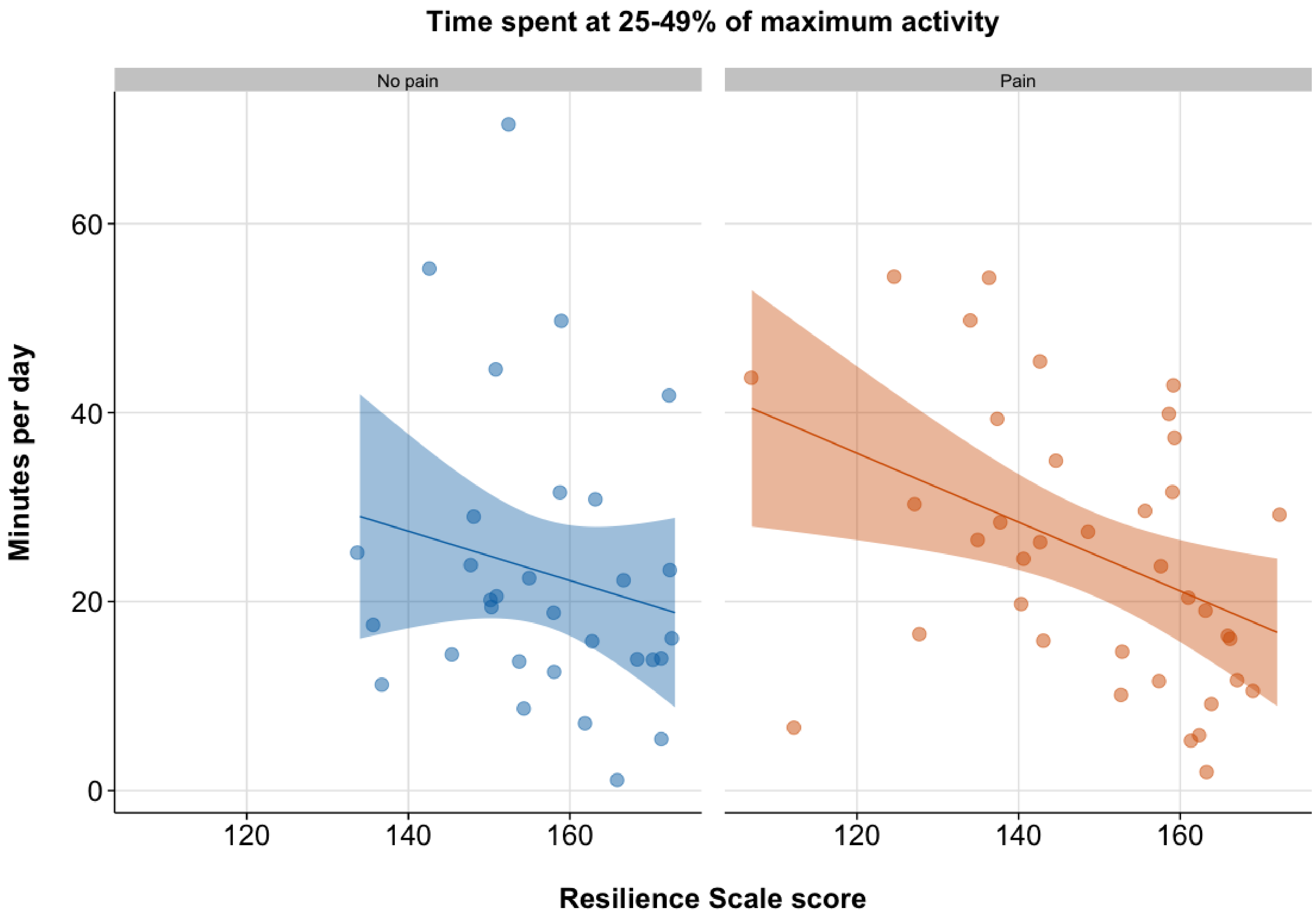
Time spent at 25-49% of maximum activity
No pain
Pain
Minutes per day
0
20
40
60
120
140
160
120
140
160
Resilience Scale score

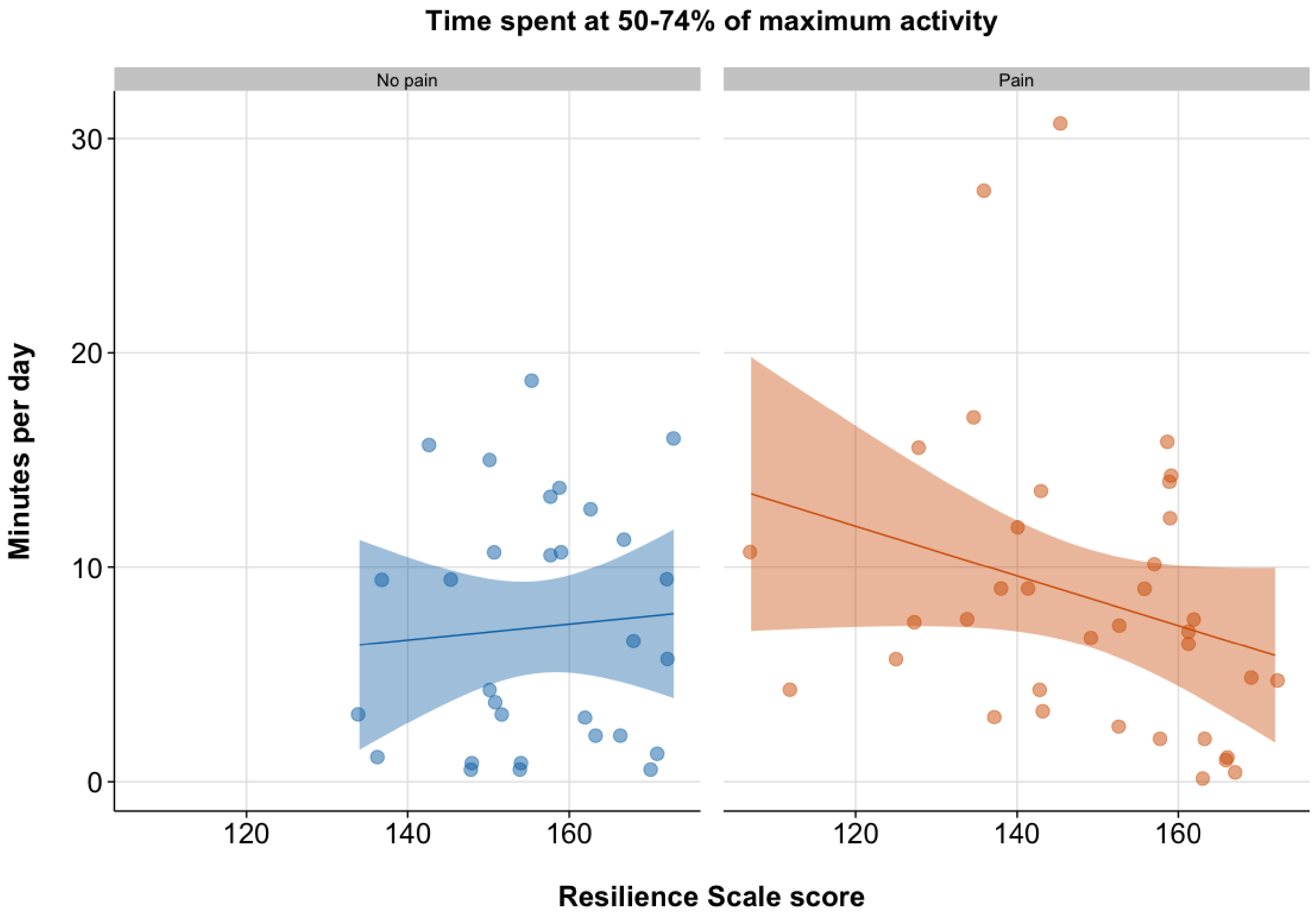

Time spent at 50-74% of maximum activity
No pain
Pain
Minutes per day
0
10
20
30
120
140
160
120
140
160
Resilience Scale score

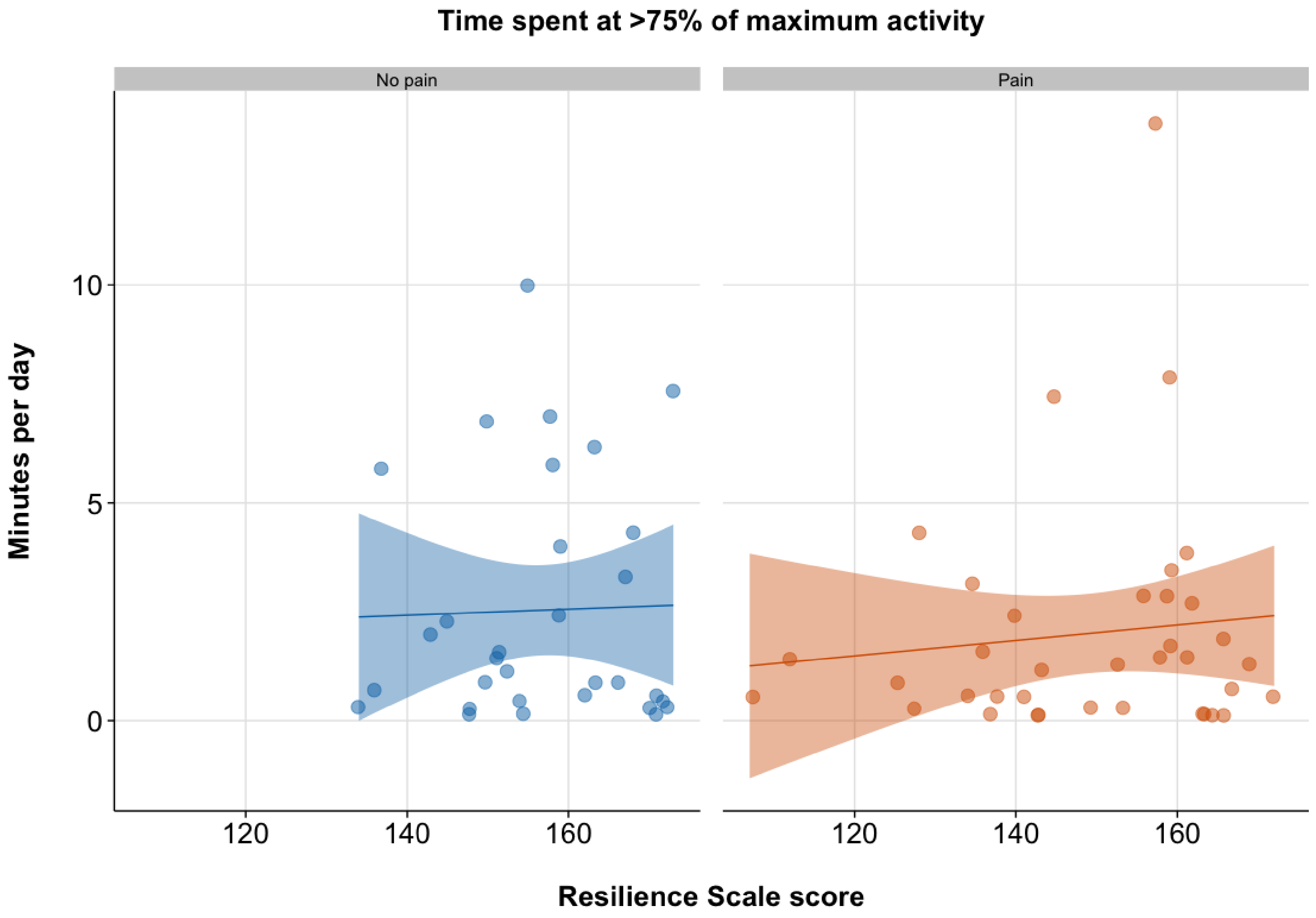

Time spent at >75% of maximum activity
No pain
Pain
Minutes per day
10
5
0
120
140
160
120
140
160
Resilience Scale score
