# Supplementary material for: Resilience does not explain the dissociation between chronic pain and physical activity in South Africans living with HIV
Source: PeerJ. 2016 Sep 13;4:e2464. doi: 10.7717/peerj.2464 (PMC5028784; doi:10.7717/peerj.2464)
Supplement: Supplemental Information 5 — No patients reported having moderate or severe difficulties with mobility on the EQ5D. [file peerj-04-2464-s005.pdf]

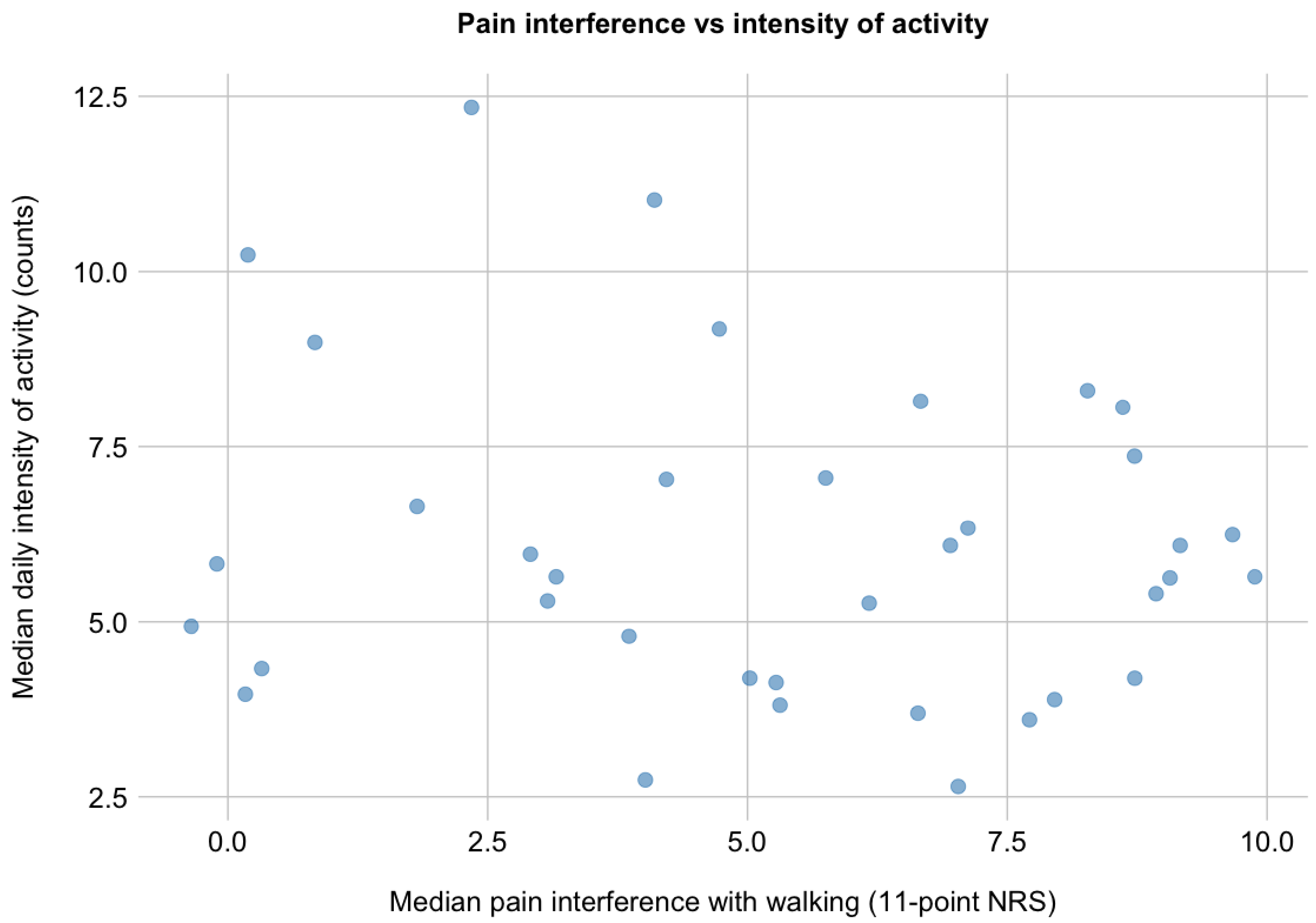

Pain interference vs intensity of activity
12.5
10.0
7.5
5.0
2.5
Median daily intensity of activity (counts)
0.0
2.5
5.0
7.5
10.0
Median pain interference with walking (11-point NRS)

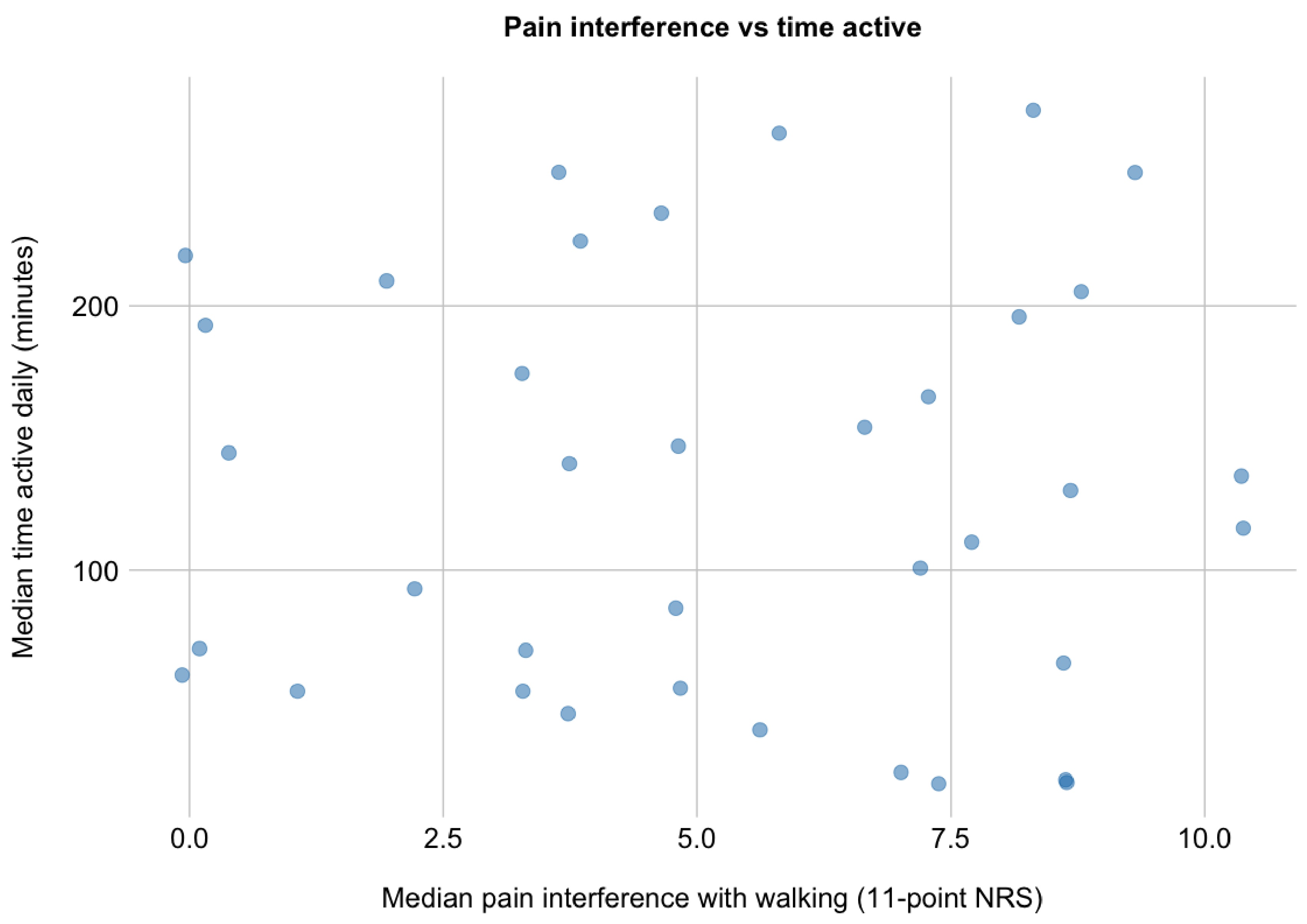
Pain interference vs time active
Median time active daily (minutes)
200
100
0.0
2.5
5.0
7.5
10.0
Median pain interference with walking (11-point NRS)

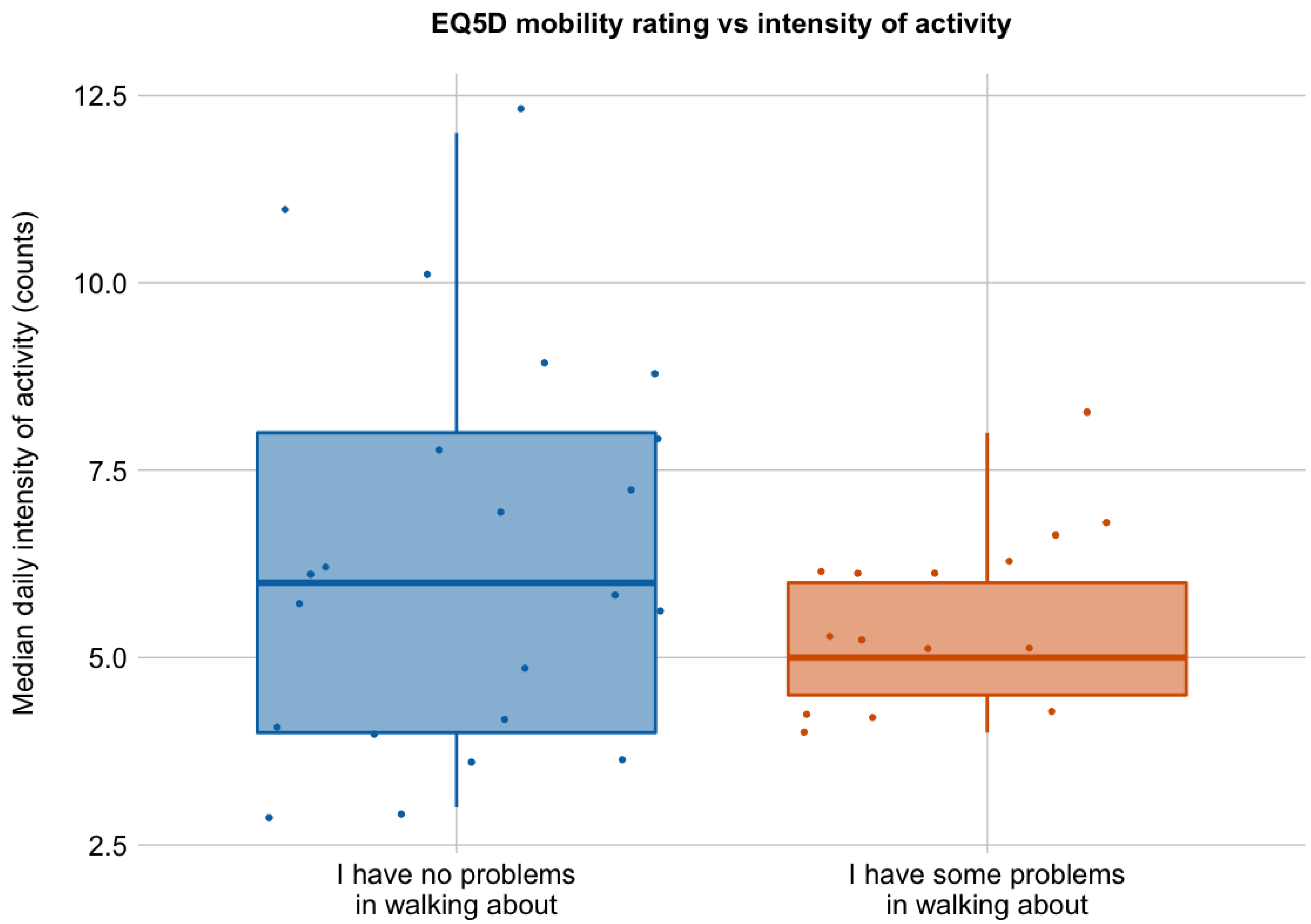
EQ5D mobility rating vs intensity of activity
Median daily intensity of activity (counts)
12.5
10.0
7.5
5.0
2.5
I have no problems
in walking about
I have some problems
in walking about

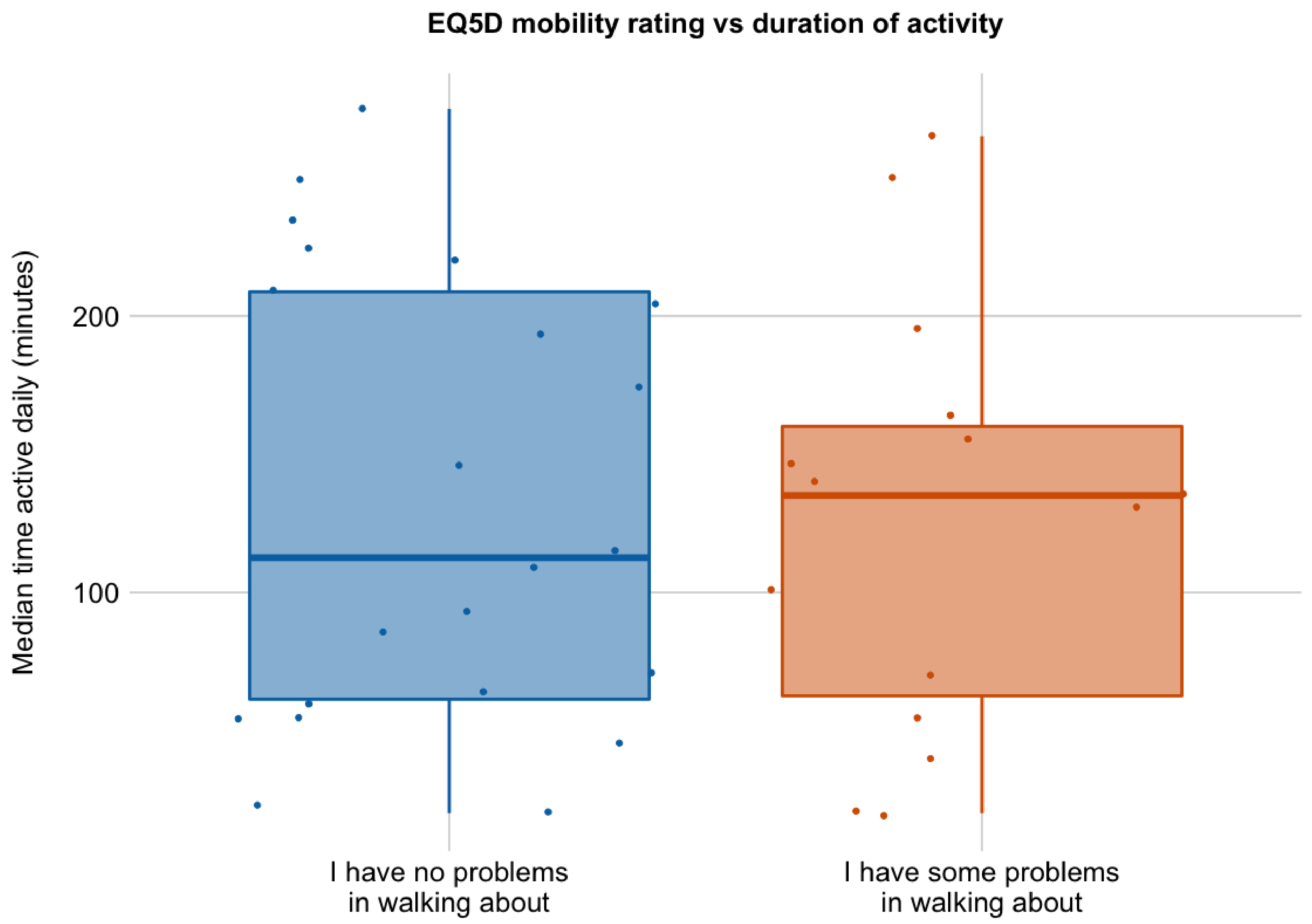
EQ5D mobility rating vs duration of activity
Median time active daily (minutes)
200
100
I have no problems in walking about
I have some problems in walking about

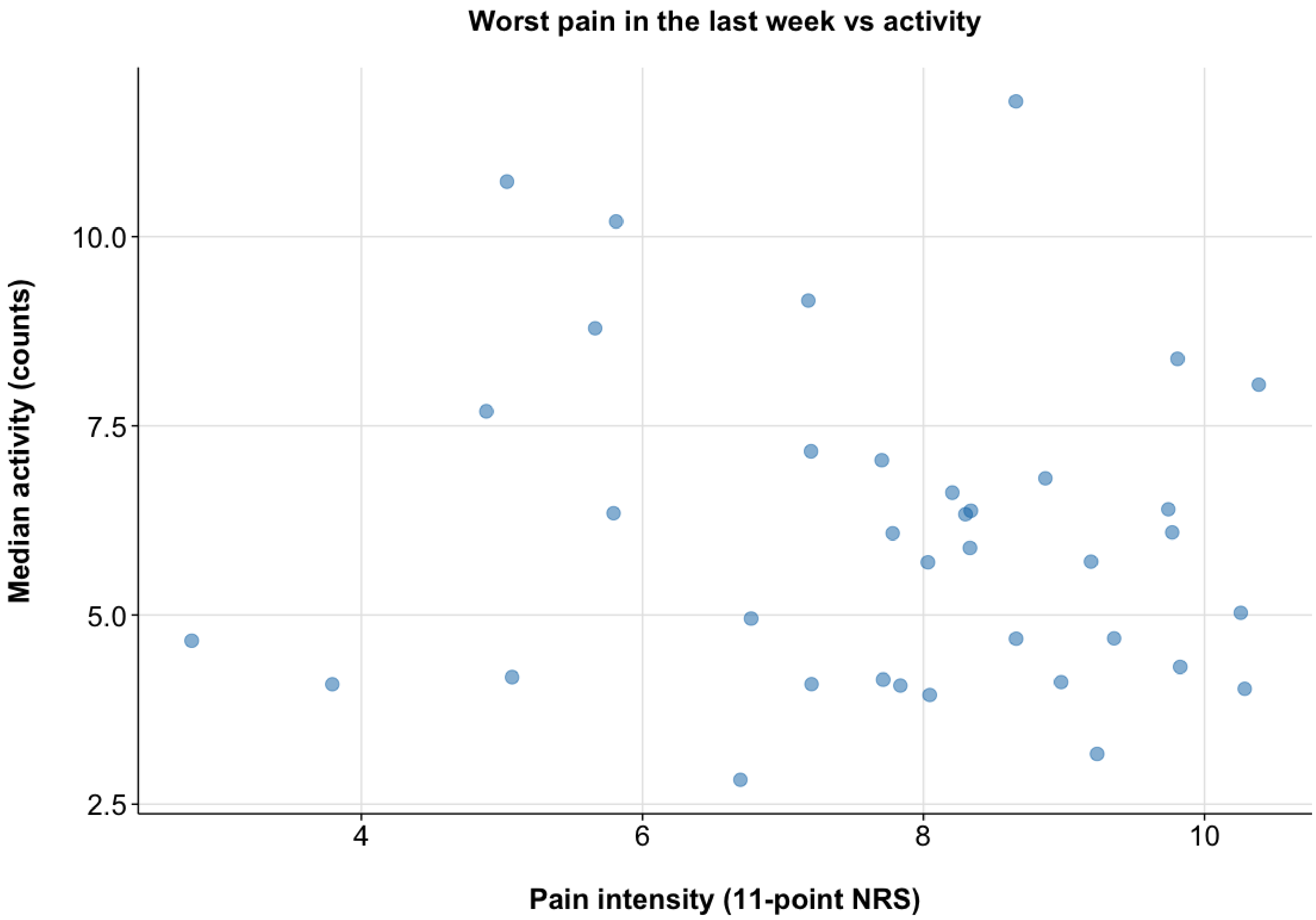

Worst pain in the last week vs activity
Median activity (counts)
10.0
7.5
5.0
2.5
4
6
8
10
Pain intensity (11-point NRS)

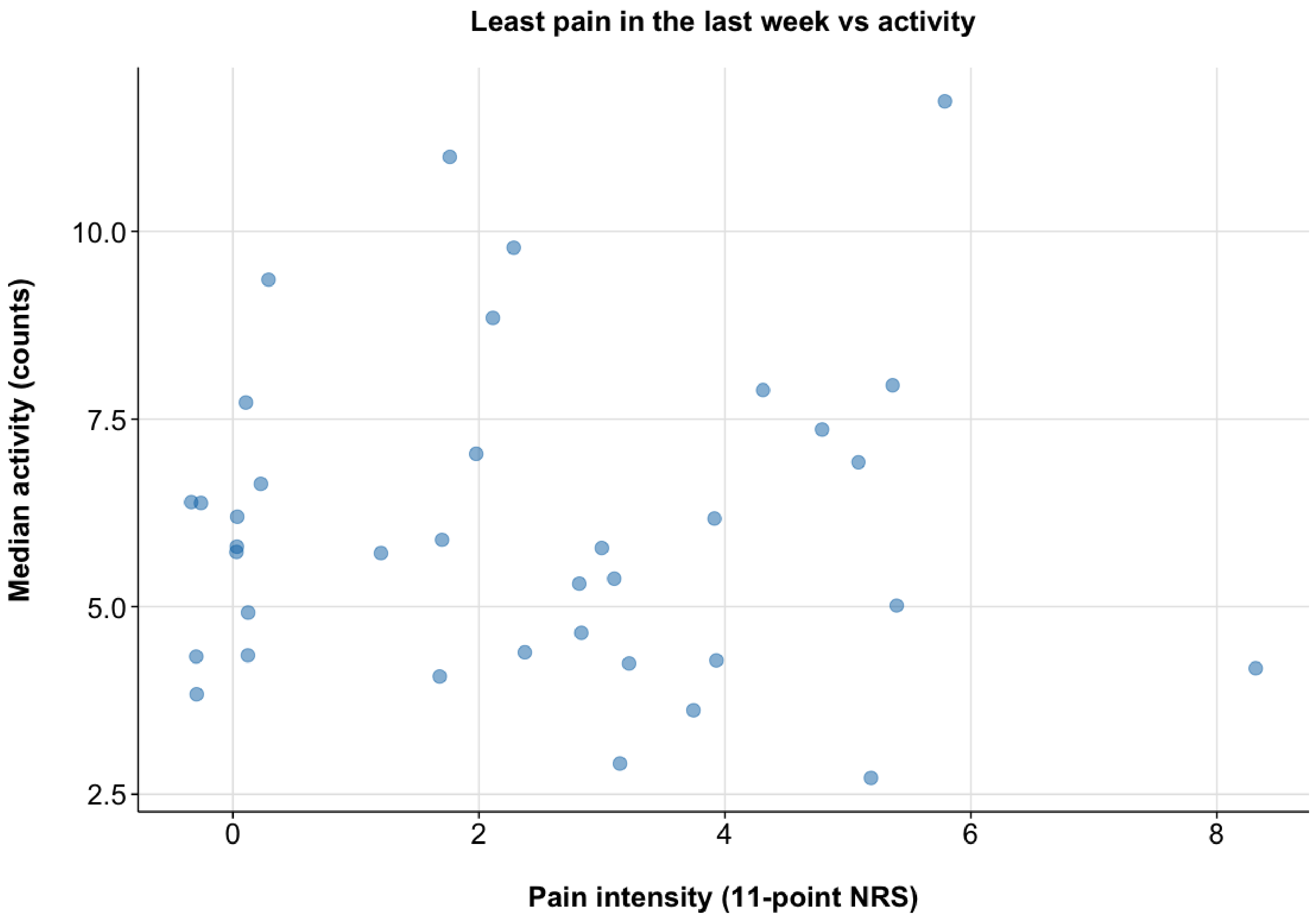

Least pain in the last week vs activity
Median activity (counts)
10.0
7.5
5.0
2.5
0
2
4
6
8
Pain intensity (11-point NRS)
